# [1,2,4]triazolo[3,4-b][1,3,4]thiadiazole derivatives as new therapeutic candidates against urease positive microorganisms: design, synthesis, pharmacological evaluations, and in silico studies

**DOI:** 10.1038/s41598-023-37203-z

**Published:** 2023-06-22

**Authors:** Minoo Khalili Ghomi, Milad Noori, Mohammad Nazari Montazer, Kamiar Zomorodian, Navid Dastyafteh, Somayeh Yazdanpanah, Mohammad Hosein Sayahi, Shahrzad Javanshir, Abbas Nouri, Mehdi Asadi, Hamid Badali, Bagher Larijani, Cambyz Irajie, Aida Iraji, Mohammad Mahdavi

**Affiliations:** 1 Endocrinology and Metabolism Research Center, Endocrinology and Metabolism Clinical Sciences Institute, Tehran University of Medical Sciences, Tehran, Iran; 2 Stem Cells Technology Research Center, Shiraz University of Medical Sciences, Shiraz, Iran; 3 Department of Medical Mycology and Parasitology, School of Medicine, Shiraz University of Medical Sciences, Shiraz, Iran; 4 Pharmaceutical and Heterocyclic Chemistry Research Laboratory, Department of Chemistry, Iran University of Science and Technology, Tehran, 16846-13114 Iran; 5 Department of Chemistry, Payame Noor University (PNU), P.O. Box 19395-3697, Tehran, Iran; 6 Department of Medicinal Chemistry, School of Pharmacy-International Campus, Iran University of Medical Science, Tehran, Iran; 7 Department of Molecular Microbiology & Immunology, and South Texas Center for Emerging Infectious Diseases, The University of Texas at San Antonio, San Antonio, TX USA; 8 Department of Medical Biotechnology, School of Advanced Medical Sciences and Technologies, Shiraz University of Medical Sciences, Shiraz, Iran; 9 Central Research Laboratory, Shiraz University of Medical Sciences, Shiraz, Iran

**Keywords:** Chemical biology, Drug discovery

## Abstract

Regarding the important role of the urease enzyme as a virulence factor in urease-positive microorganisms in this study, new series of [1,2,4]triazolo[3,4-b][1,3,4]thiadiazole derivatives were designed and synthesized. All compounds evaluated against urease enzyme exhibiting IC_50_ values of 0.87 ± 0.09 to 8.32 ± 1.21 µM as compared with thiourea as the positive control (IC_50_ = 22.54 ± 2.34 µM). The kinetic evaluations of **6a** as the most potent derivative recorded a competitive type of inhibition. Molecular dynamic simulations of the **6a** derivative were also conducted, showing that **6a** occupied the active site with closed state. Antimicrobial activities of all derivatives were performed, and **6f** (R = 3-Cl), **6g** (R = 4-Cl), and **6h** (R = 3,4-diCl) analogs demonstrated significant antifungal activities with MIC values of 1, 2, and 0.5 µg/mL compared with fluconazole with MIC = 2 µg/mL. Synthesized analogs also exhibited potent urease inhibitory activities against *C. neoformans* (IC_50_ = 83.7–118.7 µg/mL) and *P. mirabilis* (IC_50_ = 74.5–113.7 µg/mL), confirming their urease inhibitory potential. The results demonstrated that the designed scaffold could be considered a suitable pharmacophore to develop potent urease inhibitors.

## Introduction

Urease (EC 3.5.1.5) is the first known nickel-containing enzyme found in a wide variety of plants, algae, fungi, and bacteria that catalyzes the hydrolysis of urea to carbamic acids which are further hydrolysis to ammonia and carbon dioxide^1,2^. Urease is known as a virulence factor found in various pathogenic microorganisms in which the increase in the pH in the medium caused by the accumulation of NH_3_ results in the development of urolithiasis, pyelonephritis, hepatic encephalopathy, hepatic coma urolithiasis, and urinary catheter encrustation^3–5^. Also, the urease activity of *Helicobacter pylori* results in the survival of this Gram-negative bacterial in an acidic environment, such as the stomach (pH = 1–2), which plays an important role in the pathogenesis of gastric and peptic ulcer and increases the risk of gastric adenocarcinoma and gastric lymphoma^6,7^. Importantly, the urease knockout mutants can't colonize in the stomach, suggesting urease's critical role in the survival of *H. pylori* in the stomach^8,9^. In agriculture, high urease activity releasing abnormally large amounts of ammonia causes significant environmental and economic problems^10^.

**Figure 1:**
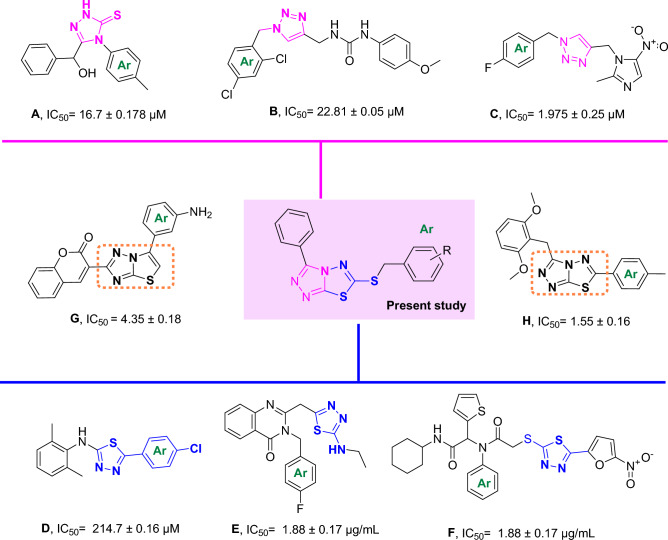
Rationalization of the newly synthesized [1,2,4]triazolo[3,4-b][1,3,4]thiadiazole derivatives with already reported triazole and thiadiazole analogs.

As a result, inhibition of urease activity can be regarded as a favorable strategy to mitigate the negative effect of ureolytic microorganisms. Triazole ring is known as a unique pharmacophore in several pharmaceuticals and natural products as antimalarial^11^, antiviral^12^, antibacterial^13^ as well as antifungal agents^14^. Triazole rings' favorable properties, including moderate dipole properties, hydrogen bonding capability, and rigidity, are responsible for their enhanced biological activities^15,16^. Triazole-containing derivatives show promising anti-urease activities that prevent the drug's resistance and have attracted great interest in searching for new anti-urease agents^17,18^. Compounds **A**^19^, **B**^20^ and **C**^21^ (Fig. 1) are some reports of triazole derivatives with inhibitory potencies against urease.

The biological activities of various thiadiazole derivatives and their N-bridged heterocyclic analogs have also been extensively studied. Regarding that thiadiazole is the bioisostere of pyrimidine and oxadiazole^22^, analogous of this moiety exhibits a wide range of pharmacological properties, including antiviral, antibacterial, antifungal, antiparasitic, anti-inflammatory, and anticancer activities^23^. More importantly, its relative lipophilicity attributed to the presence of the sulfur atom provides the optimum situation to cross the cellular membranes and induce its effect^24^.

Khan et al. reported a series of 2,5-disubstituted-1,3,4-thiadiazoles as urease inhibitors, and compound **D** was found to be the best potencies among thiadiazole analogs^25^. In 2019, the urease inhibitory activities of triazole, thiadiazole, and thiosemicarbazide linked to quinazoline-4(3H)-one was evaluated, and among them, compound bearing thiadiazole (**E**, Fig. 1) recorded the best urease inhibitory potencies with IC_50_ = 1.88 ± 0.17 µg/mL. According to their in silico study, thiadiazole ring effectively interacted with the urease binding site^26^. In a recent study, the most potent derivative of 5-nitrofuran-2-yl-thiadiazole derivative (**F**, Fig. 1) showed a tenfold improvement in the inhibitory potency against urease compared to thiourea as a positive control with the non-competitive mode of inhibition^27^.

Triazolo-thiadiazole is an interesting heterocyclic compound synthesized by fusing 1,2,4-triazole and 1,3,4-thiadiazole rings. Triazolo-thiadiazole is an important analog in many biologically active compounds that showed antibacterial, antifungal, and anti-inflammatory activities^28,29^. However, limited research was conducted on the anti-urease properties of triazole-thiadiazole derivatives, and compounds **G**^30^ and **H**^31^ (Fig. 1) are a few examples with improved inhibition compared to the parental structures.

As a result, in the current study, new series of novel triazole-thiadiazole were designed, synthesized, and the anti-urease properties of newly designed compounds were examined against *Jack bean* urease. Next, the antimicrobial, as well as antiurease properties of these derivatives were evaluated against urease-positive microorganisms. Also, the molecular dynamics simulations of the most potent derivative were performed to get an insight into its behavior within the binding site.

## Results and discussion

### Chemistry

**Scheme 1:**
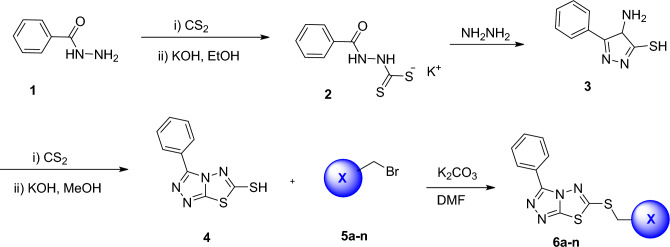
Synthesis of [1,2,4]triazolo[3,4-b][1,3,4]thiadiazole derivatives **6a**–**n**.

Synthesis of the title compounds **6a**–**o** was schematically described in Scheme 1. It was initiated by the reaction of carbon disulfide and benzohydrazide (compound **1**) in ethanol in the presence of catalytic amounts of potassium hydroxide. The reaction mixture was stirred for 16 h at room temperature. The product was filtered off and washed with ether to remove excess carbon disulfide. Next, compound **2** and hydrazine monohydrate was refluxed for 4 h in water. The mixture was cooled to room temperature, diluted with water and the white solid of the required triazole was precipitated out. Next to the solution of 4-amino-5-phenyl-4H-pyrazole-3-thiol (compound **3**) in MeOH, potassium hydroxide was added, and the mixture was sired at room temperature for 30 min, followed by dropwise addition of carbon disulfide at 5 °C, and then reflux at 70 °C for 8–12 h. Finally, aryl halides (compound **5a**–**n**) were added to the 3-phenyl-[1,2,4]triazolo[3,4-b][1,3,4]thiadiazole-6-thiol (compound **4**) in DMF in the presence of potassium carbonate at room temperature. The structure of final products **6a**–**n** was confirmed using NMR, IR, ESI–MS, and elemental analysis.

### Urease inhibition and structure–activity relationships

All newly synthesized compounds (**6a**–**n**) were examined for their inhibitory potentials against the urease enzyme, the results were presented in Table 1, and the structure–activity relationships (SARs) were constructed. From the experimental data, it appears that all of the synthesized compounds have a significant inhibitory effect with IC_50_ values in the range of 0.87 ± 0.09 to 8.32 ± 1.21 µM as compared with thiourea as the positive control (IC_50_ = 22.54 ± 2.34 µM).

**Table 1:** The urease inhibitory activities of **6a**–**o** in the enzymatic assessments.


Compound	R	IC_50_ (µM) ± SD
**6a**	H	0.87 ± 0.09
**6b**	2-F	1.51 ± 0.21
**6c**	3-F	1.40 ± 0.29
**6d**	4-F	1.01 ± 0.13
**6e**	2-Cl	8.32 ± 1.21
**6f**	3-Cl	1.25 ± 0.19
**6g**	4-Cl	1.68 ± 0.73
**6h**	3,4-diCl	1.41 ± 0.32
**6i**	2-Br	1.59 ± 0.14
**6j**	3-Br	1.05 ± 0.34
**6k**	4-Br	1.53 ± 0.17
**6l**	4-NO_2_	2.22 ± 0.18
**6m**	2-CH_3_	1.60 ± 0.23
**6n**	3-CH_3_	1.23 ± 0.56
**Thiourea**^**a**^		22.54 ± 2.34

Data presented here are the mean ± SD of three independent experiments.

^a^Positive control.

The parent compound **6a** exhibited the best inhibitory activity against urease with an IC_50_ value of 0.87 µM. This derivative showed around 27 times improvement in the activity *vs* positive control.

Evaluation of the fluorine derivatives as halogen-substituted analogs revealed that they exhibited good potencies against urease, with no significant differences observed among them. However, the 4-F derivative (**6d**) demonstrated better results with an IC_50_ value of 1.01 µM. It was followed by the 3-F derivative (**6c**) with an IC_50_ value of 1.40 µM, and the 2-F derivative (**6b**) with an IC_50_ value of 1.51 µM. These findings indicate that the presence of fluorine substitutions enhances the inhibitory activity against urease, and the position of the fluorine atom can influence the potency of the compound.

Detailed evaluations of the chlorine-substituted compounds revealed that the 2-Cl derivative (**6e**) exhibited inferior activity and recorded the worst results within this group, with an IC_50_ value of 8.32 µM. However, it is worth noting that even though this derivative had reduced potency, it still displayed better inhibitory activity compared to the positive control. Interestingly, when the position of the chlorine atom was changed from *ortho* to *meta* (**6f**) and *para* (**6g**), potency was significantly improved. The IC_50_ values for these derivatives were 1.25 µM and 1.68 µM, respectively, indicating a substantial enhancement in inhibitory activity. Additionally, the compound with 3,4-diCl substitution (**6h**) exhibited an IC_50_ value of 1.41 µM, further demonstrating its potent inhibitory effect against urease. These results suggest that the position and number of chlorine substitutions play a crucial role in modulating the potency of the compounds, with meta and para positions leading to improved activity compared to the ortho position.

In the cases of bromine derivatives as a large electron withdrawing group (**6i**–**k**), 3-Br compounds showed the best result (IC_50_ = 1.05 µM) followed by 4-Br ≥ 2-Br. Substitution of 4-nitro moiety (**6l**) as hydrophilic and high electron withdrawing group at R position deteriorated the potencies compared to all derivatives except **6e**.

In comparison to the electron-withdrawing group, compounds containing electron-donating moieties (**6m** and **6n**) also exhibited good potency against urease. Similar to the previous derivatives, no statistical differences were observed between the *ortho* and *meta* analogs. These findings suggest that introducing electron-donating groups can contribute to good inhibitory activity against urease, and the position of these groups did not significantly affect the potency.

Literature reviews were conducted to assess the trend of SAR in the current study concerning previously reported articles. A study with the thiazolidinone derivatives showed that although the main backbone is active, electron-donating groups exhibited slightly superior activity^32^. Another study indicated that the branched analogs of thiazolidine ester are less active than their straight-chain counterparts, potentially due to the steric bulk of the branched-chain substituents^33^. In another study evaluating the anti-urease potency of coumarin-thiazolotriazole, although a straightforward SAR analysis was not reported, the following trend was observed: 3-NH_2_ > 3-MeO-4-OH > phenyl^34^. Additionally, diindolylmethane bearing thiadiazole was designed as a potent urease inhibitor. The study reported that the backbone itself exhibited high potency against urease, and the SAR analysis demonstrated that analogs with electron-withdrawing groups on the phenyl ring displayed greater potential compared to analogs with electron-donating groups^35^.

Considering that the basic structure of the compounds is somewhat different, it is impossible to extract a general rule, but it is clear that the presence of triazolo-thiadiazole has a positive effect on the anti-urease potency and further studies have to be conducted to comprehensively extract the SAR. Overall, in the current study, the highest potency was observed in the unsubstituted derivative **6a**, suggesting that, similar to the previous study, the designed backbone possesses considerable inhibitory activity against urease. In most cases, no significant differences were observed between the types and positions of substituted moieties. However, one exception to this trend was observed in **6e** with an IC_50_ value of 8.32 µM. The results support our hypothesis that the designed backbone exhibits high potency, regardless of the specific type of substitutions. This emphasizes the robustness and effectiveness of the designed structure as a potential inhibitor of urease.

### Enzyme kinetic studies

**Figure 2:**
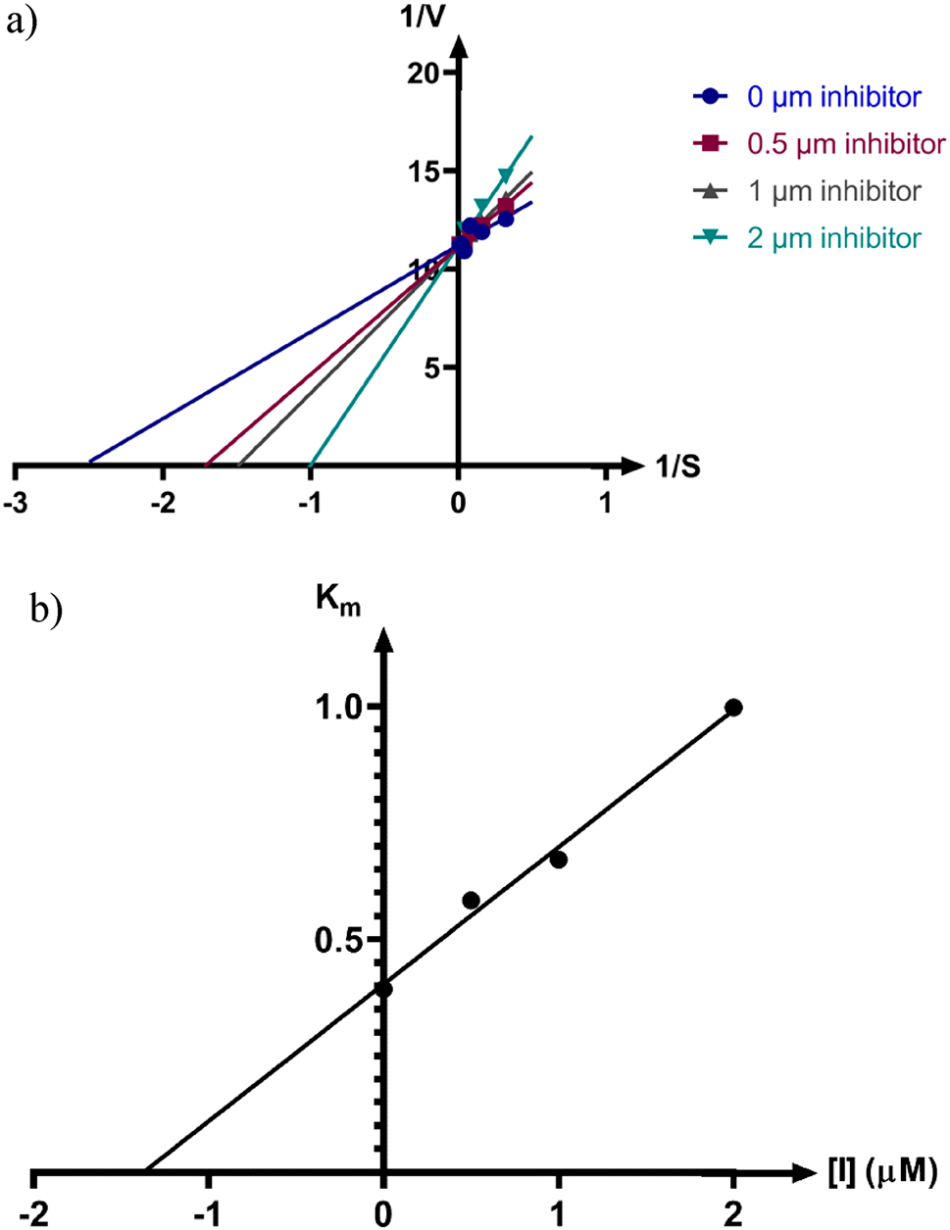
Kinetics of urease inhibition by **6a**. (**a**) The Lineweaver–Burk plot in the absence and presence of different concentrations of sample **6a**; (**b**) The secondary plot between *K*_m_ and various concentrations of **6a**.

According to Fig. 2a, the Lineweaver–Burk plot showed that **6a** is a competitive-type inhibitor. Furthermore, the plot of the *K*_m_ versus different concentrations of inhibitor gave an estimate of the inhibition constant, *K*_i_ of 1.37 µM (Fig. 2b).

### Molecular dynamics study

**Figure 3:**
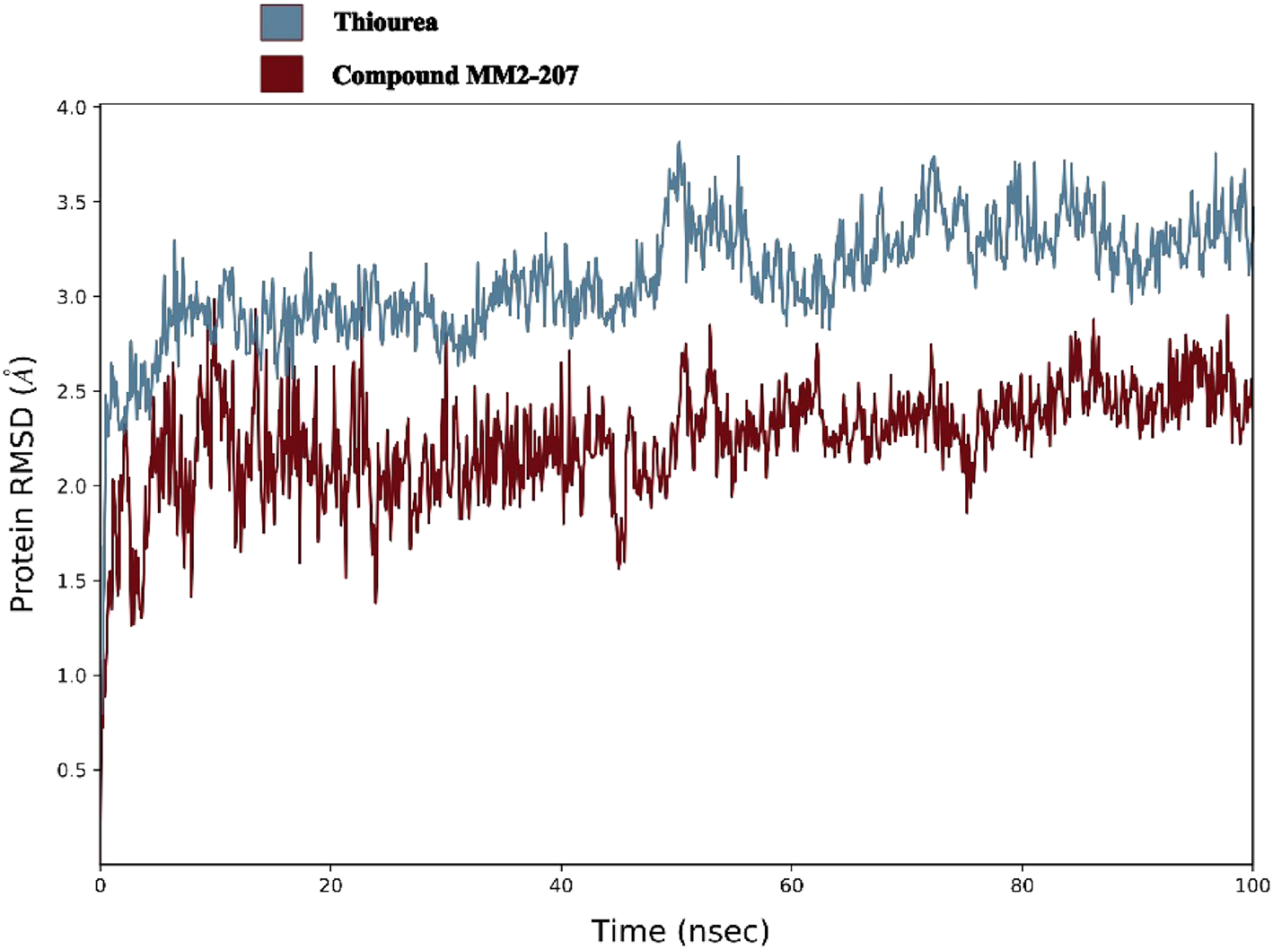
The RMSD of *Jack bean* urease in complex with thiourea (blue) and compound **6a** (red).

The molecular dynamic study was conducted based on the comparison between *Jack bean* urease in complex with thiourea as the natural physiologic ligand and in complex with compound **6a** at the most potent synthesized compound. The root means square deviation (RMSD) of backbone atoms is an indicator of complex steadiness, the RMSD of both complexes during the simulation course of 100 ns showed in Fig. 3.

As shown in Fig. 3, the simulation time was adequate for both complexes to reach acceptable steadiness (fluctuations under 3 Å). Moreover, the deviation value of compound **6a** complex was notably lower than the thiourea complex, which could be interpreted as the higher stability of *Jack bean* urease in complex with the synthesized compound rather than the natural state.

**Figure 4:**
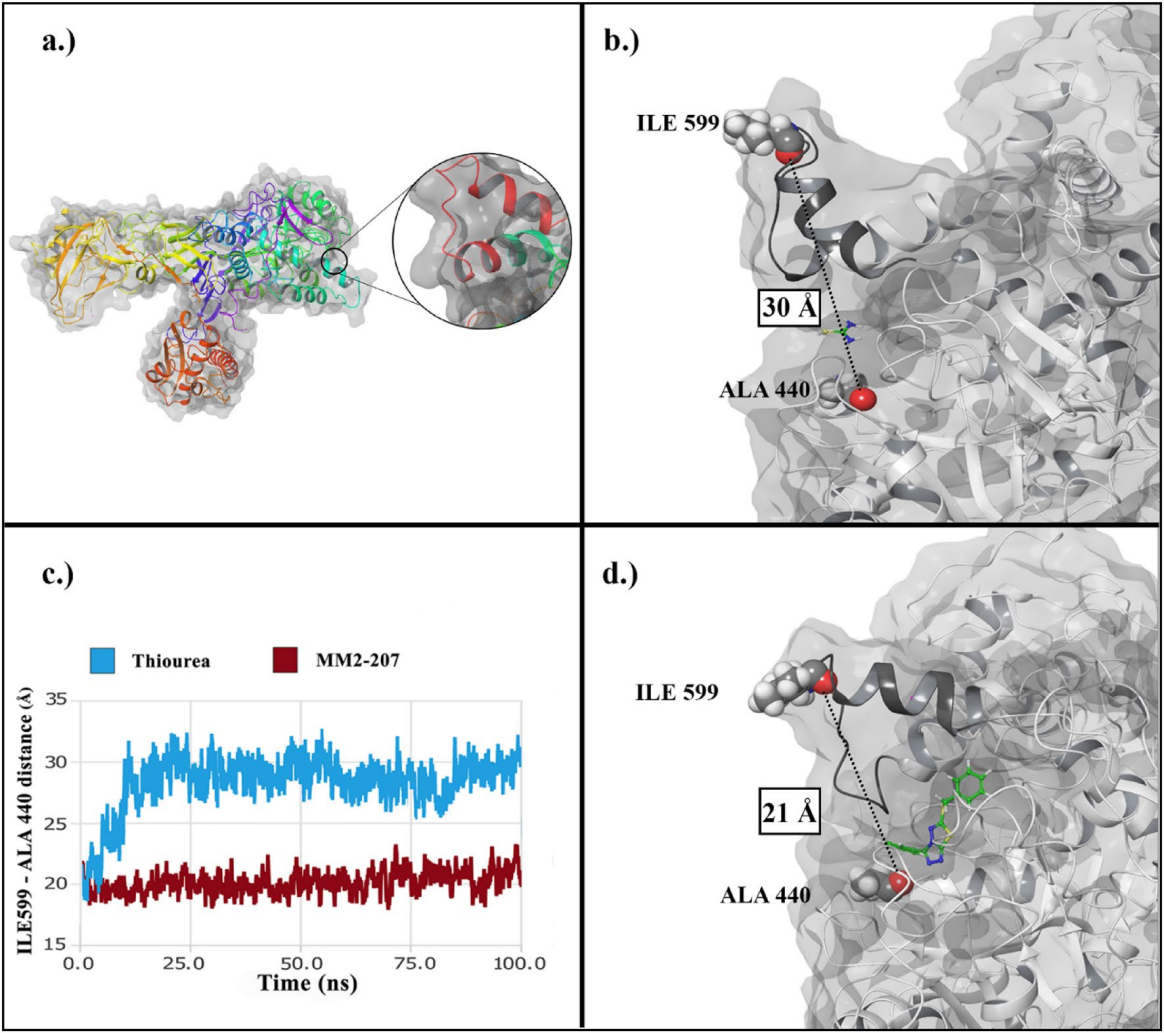
(**a**) *Jack bean* urease structure and the dynamic flap of the active site pocket (red). (**b**) The open state of the flap (black) in complex with thiourea (**c**) The comparison of flap distance through simulation time for thiourea (blue) and **6a** (red) complexes (**d**) The closed state of the flap (black) in complex with **6a**.

The quaternary structure of *Jack bean* urease enzyme involves four subunits, the (αβ)_8_ TIM barrel domain is the enzyme's active site, which consists of two nickel ions coordinated by the unusual amino acid KCX490. Other coordinating residues such as His407, His409, Asp633, KCX490, His519, His545, and Gly550 are vital for the urease enzyme activity. Based on previous studies^36^, the hydrolysis stage of urea depends on residues Met590–His607. These residues form a helix-turn-helix shape, a dynamic part of the active site pocket called the mobile flap (Fig. 4a). The closed state of mobile flap conformation can restrict access to the active site pocket of the enzyme. The distance between Ile599-Ala440 residues could indicate the active site flap. As demonstrated in Fig. 4, the average distance for compound **6a** was 21 Å (close state) compared to thiourea with 30 Å (open state).

**Figure 5:**
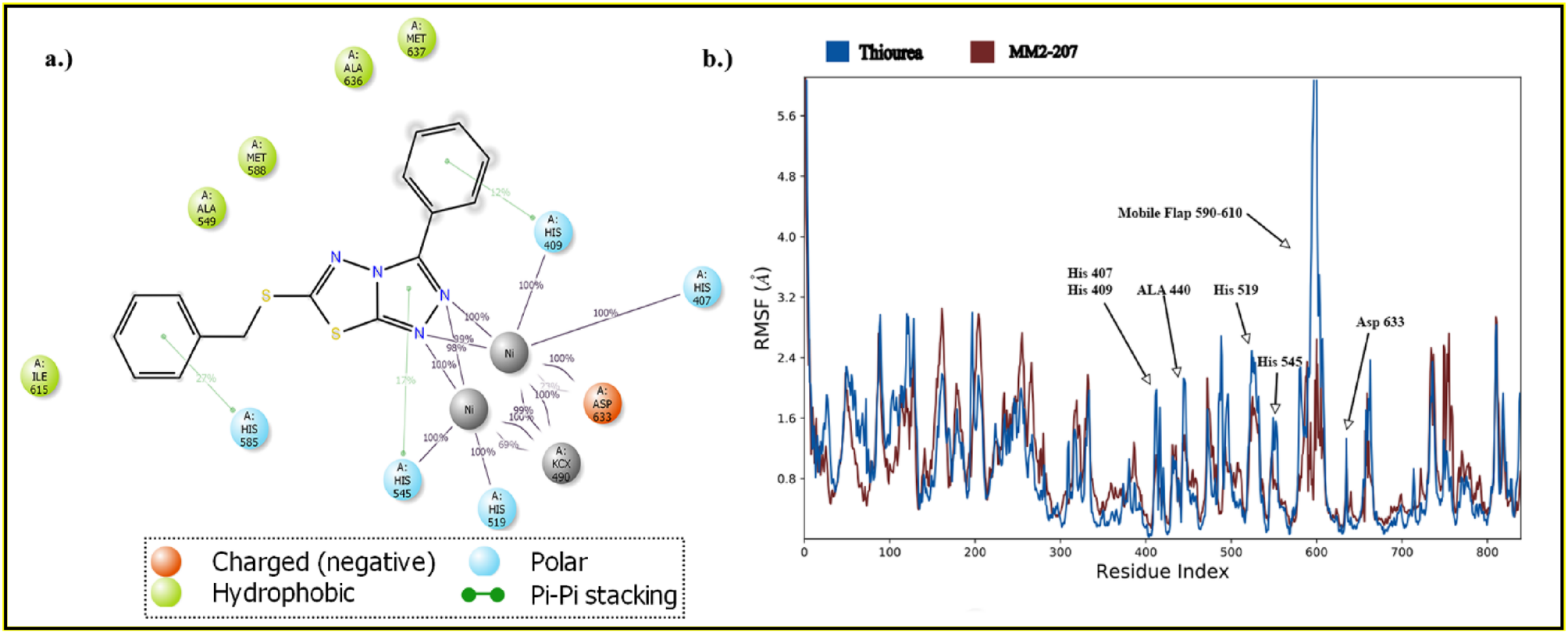
(**a**) 2D interactions of compound **6a** with the active site of *Jack bean* urease (**b**) RMSF of thiourea complex (blue) and **5a** (red).

Root mean square fluctuation (RMSF) displays the fluctuations of backbone atoms and can be interpreted as a mark of stability for residues that have a smaller value (Fig. 5). The strength of interacting residues with compound **6a** correlated with the RMSF of these residues compared with corresponding residues in the thiourea complex. Mobile flap residues (590–610) notably showed lower fluctuation in **6a** complex. Furthermore, other interacting residues, His407, His409, Ala440, His519, His545, and Asp633, showed less fluctuation in the **6a** complex compared with the thiourea complex.

### Antimicrobial activity and structure–activity relationships study

The antimicrobial activity of the synthesized compounds **6a**–**n** was determined according to the Clinical and Laboratory Standards Institute (CLSI) methods against *Cryptococcus neoformans* (H99) and *Proteus mirabilis*. Table 2 shows the minimum inhibitory concentrations (MICs) and minimum fungicidal concentrations (MFCs) values of the target compounds compared with fluconazole and ciprofloxacin as the standard drug.

**Table 2:** The antibacterial activity of compounds **6a**–**n.**

Microorganism	Compound	MIC (µg/mL)	MFC/MBC (µg/mL)
*Cryptococcus neoformans* (H99)	**6a**	> 256	ND
	**6b**	> 256	ND
	**6c**	> 256	ND
	**6d**	> 256	ND
	**6e**	> 256	ND
	**6f**	1	2
	**6g**	2	4
	**6h**	0.5	0.5
	**6i**	> 256	ND
	**6j**	> 256	ND
	**6k**	> 256	ND
	**6l**	> 256	ND
	**6m**	> 256	ND
	**6n**	> 256	ND
	**Fluconazole**	2	–
*Proteus mirabilis*	**6a**	256	ND
	**6b**	256	ND
	**6c**	256	ND
	**6d**	256	ND
	**6e**	256	ND
	**6f**	256	ND
	**6g**	256	ND
	**6h**	256	ND
	**6i**	256	ND
	**6j**	256	ND
	**6k**	256	ND
	**6l**	256	ND
	**6m**	256	ND
	**6n**	256	ND
	**Ciprofloxacin**	0.25	–

*MIC* minimum inhibitory concentration, *MFC* minimum fungicidal concentration, *MBC* minimum bactericidal concentration, *ND* not determined.

Assessment against *C. neoformans* yeast exhibited that just chlorine-containing derivatives, including **6f** (R = 3-Cl), **6g** (R = 4-Cl), and** 6h** (R = 3,4-diCl) derivatives, recorded significant antifungal activities with MIC values of 1, 2, and 0.5 µg/mL, respectively compared with fluconazole with MIC value of 2 µg/mL. These derivatives also exhibited promising MFC with values of 2, 4, and 0.5 µg/mL. Similar to enzymatic assessments, **6e** entry bearing 2-Cl substitution was almost inactive under the tested concentrations. It was understood that the presence of chlorine substitutions at *meta* and *para* portions of the benzyl ring significantly improved the fungicidal activities of the designed scaffold.

Evaluations of the Gram-negative bacterium *P. mirabilis* showed that all derivatives behaved similarly and demonstrated MIC values of 256, confirming the backbone's potencies regardless of the type of derivitizations.

### Urease inhibitory activities against urease-positive microorganisms

To properly evaluate the mechanism of **6a**–**n** derivatives, their potencies to reduce the urease activities were also assessed against urease-positive microorganisms (Table. 3). The results were reported in terms of IC_50._ Evaluation against *C. neoformans* revealed that the unsubstituted analog had an IC_50_ value of more than 123.7 µg/mL. Any substitutions on the benzyl ring were in favor of inhibition. Assessments of the halogen-substituted groups at different positions exhibited that fluorine substitution at any position of the benzyl ring (**6b**, **6c**, and **6d**) as a small and strong electron withdrawing group slightly improved the potencies *vs* **6a**. Noteworthy, **6e**, **6f,** and **6g** containing chlorine group exhibited promising potency with IC_50_ values of 101.9, 87.5, and 93.3 µg/mL, respectively. Multi-substitution of chlorine (**6h**, IC_50_ = 83.7 µg/mL) also improved potency compared to mono-substituted derivatives.

**Table 3:** The urease inhibitory activities of **6a**–**o** in the term of IC_50_ (µg/mL) ± SD against urease-positive microorganisms.

Compound	*C. neoformans*	*P. mirabilis*
**6a**	123.7 ± 9.5	78.2 ± 8.5
**6b**	111.1 ± 7.4	87.3 ± 4.9
**6c**	117.4 ± 6.4	79.8 ± 3.5
**6d**	117.8 ± 8.6	91.0 ± 6.4
**6e**	101.9 ± 3.3	93.3 ± 5.2
**6f**	87.5 ± 8.1	73.2 ± 5.5
**6g**	94.3 ± 5.9	75.4 ± 6.1
**6h**	83.7 ± 3.2	74.5 ± 4.7
**6i**	112.2 ± 6.7	109.4 ± 6.2
**6j**	111.4 ± 4.8	112.3 ± 6.9
**6k**	118.7 ± 5.5	113.7 ± 4.6
**6l**	113.6 ± 5.4	91.8 ± 7.0
**6m**	110.9 ± 3.8	95.7 ± 6.6
**6n**	112.3 ± 4.6	96.8 ± 4.7

Values represent means ± SD of 3 independent experiments.

Compounds **6i**, **6j**, and **6k** containing bromine as a large electron withdrawing group did not demonstrate significant differences compared to chlorine counterparts. The same trend was seen in the electron-donating group, which slightly improved the activity as compared to **6a**.

*P. mirabilis* revealed IC_50_ value in the 73.2 ± 5.5 to 113.7 ± 4.6 µg/mL range. The best results came back to **6f (**R = 3-Cl**)**,** 6h (**R** = **3,4-diCl), and **6g (**R** = **4-Cl). No significant differences exist among the other derivatives bearing fluorine, nitro, and methyl derivatives.

Further evaluations of the site of derivatization did not display statistically significant differences among *ortho, meta,* and *para* positions. Overall, the best results came back to compound **6l** bearing 3,4-diCl analog. This could be due to the favorable effects of lipophilicity and steric hindrance.

## Conclusions

In summary, in this study, a series of [1,2,4]triazolo[3,4-b][1,3,4]thiadiazole derivatives was developed and evaluated as urease inhibitors. All derivatives exhibited outstanding urease inhibition in the enzymatic assessments with IC_50_ ranging from 0.87 ± 0.09 to 8.32 ± 1.21 µM as compared with thiourea as the positive control with IC_50_ value of 22.54 ± 2.34 µM. The SARs analysis through the different types of substitutions displayed that unsubstituted compound **6a** recorded the best urease inhibition of the series. The kinetic studies of the most active compound disclosed that this compound exhibited a competitive inhibitor with *K*_i_ of 1.37 µM. MD study showed that the compound **6a** exhibited pronounced interaction with essential urease active site and mobile flap residues through the [1,2,4]triazolo[3,4-b][1,3,4]thiadiazole moiety by coordinating toward the metal bi-nickel complex. Antimicrobial activities of all analogs with CLSI methods displayed that chlorine derivatives, including **6f** (R = 3-Cl), **6g** (R = 4-Cl), and** 6h** (R = 3,4-diCl) were highly potent against *C. neoformans* with MFCs of 2, 4, and 0.5 µg/mL, respectively. On the other hand, all derivatives recorded MICs value of 256 against *P. mirabilis.* Overall, the urease activities of all derivatives against urease-positive microorganisms showed that **6h** was regarded as a potent derivative against *P. mirabilis* and *C. neoformans* with IC_50_ values of 74.5 ± 4.7 and 83.7 ± 3.2 µg/mL.

Based on the information provided, it appears that [1,2,4]triazolo[3,4-b][1,3,4]thiadiazole derivatives have shown high activity against urease enzyme and urease-positive microorganisms, indicating their potential as urease inhibitors. Additionally, in silico studies have provided supportive results. The proposed mechanism for the activity of these derivatives suggests that they might inhibit the urease enzyme. However, it is important to note that further studies are required to fully evaluate and understand the mechanism of action of these compounds.

## Material and methods

### Synthesis

#### 6-(benzylthio)-3-phenyl-[1,2,4]triazolo[3,4-b][1,3,4]thiadiazole (**6a**)

Brown solid; Yield: 81%; MP = 217–219 °C; IR (KBr, v_max_, 3030 (CH Aromatic), 2975 (CH Aliphatic), 1451,1435 (C=N) Cm^−1^; ^1^H NMR (250 MHz, DMSO-d_6_) δ 8.16 (d, *J* = 7.50 Hz, 2H, H_2_, H_6_), 7.65–7.47 (m, 5H, H Ar), 7.41–7.25 (m, 3H, H_3_,, H_4_, H_5_), 4.65 (s, 2H, CH_2_), ppm. ^13^C NMR (62 MHz, DMSO-d_6_): δ 168.36, 157.68, 146.49, 143.04, 139.25, 137.48, 135.29, 134.41, 132.06, 131.25, 130.90, 129.26, 128.93, 128.78, 128.59, 128.43, 127.56, 127.43, 127.19, 126.62, 125.23, 122.92121.36, 115.09, 36.73 ppm; ESI–MS (C_16_H_12_N_4_S_2_): calculated m/z 324.05 [M+H]^+^, observed m/z 324.08 [M+H]^+^; Anal. Calcd C_16_H_12_N_4_S_2_, C, 59.23; H, 3.73; N, 17.27; Found; C, 59.44; H, 3.95; N, 17.47.

#### 6-((2-fluorobenzyl)thio)-3-phenyl-[1,2,4]triazolo[3,4-b][1,3,4]thiadiazole (**6b**)

Brown solid; Yield: 71%; MP = 190–192 °C; IR (KBr, v_max_, 3040 (CH Aromatic), 2950 (CH Aliphatic), 1452,1438 (C=N) Cm^−1^; ^1^H NMR (250 MHz,DMSO-d_6_) δ 8.15 (d, *J* = 7.10 Hz, 2H, H_2_, H_6_), 7.61–7.51 (m, 4H, H Ar), 7.40–7.30 (m, 1H, H Ar), 7.27–7.13 (m, 2H, H Ar), 4.67 (s, 2H, CH_2_), ppm. ^13^C NMR (62 MHz, DMSO-d_6_): δ 133.72, 132.78, 132.65, 131.79, 130.59, 127.27, 126.79, 117.08, 116.74, 38.20 ppm; ESI–MS (C_16_H_11_FN_4_S_2_): calculated m/z 342.04 [M+H]^+^, observed m/z 342.15 [M+H]^+^; Anal. Calcd C_16_H_11_FN_4_S_2_, C, 56.12; H, 3.24; N, 16.36; Found; C, 56.29; H, 3.49; N, 16.61 (Supplementary file).

#### 6-((3-fluorobenzyl)thio)-3-phenyl-[1,2,4]triazolo[3,4-b][1,3,4]thiadiazole (**6c**)

Brown solid; Yield: 75%; MP = 216–218 °C; IR (KBr, v_max_, 3045 (CH Aromatic), 2975 (CH Aliphatic), 1452,1430 (C=N) Cm^−1^; ^1^H NMR (250 MHz, DMSO-d_6_) δ 8.13 (d, *J* = 6.10 Hz, 2H, H_2_, H_6_), 7.60–7.49 (m, 3H, H Ar), 7.42–7.29 (m, 3H, H Ar), 7.11 (t, *J* = 7.50 Hz, 1H, H_4_), 4.65 (s, 2H, CH_2_), ppm. ^13^C NMR (62 MHz, DMSO-d_6_): δ 132.06, 131.93, 131.78, 130.54, 127.26, 126.71, 117.58, 117.22, 116.30, 115.96, 38.32 ppm; ESI–MS (C_16_H_11_FN_4_S_2_): calculated m/z 342.04 [M+H]^+^, observed m/z 342.10 [M+H]^+^; Anal. Calcd C_16_H_11_FN_4_S_2_, C, 56.12; H, 3.24; N, 16.36; Found; C, 56.30; H, 3.45; N, 16.50.

#### 6-((4-fluorobenzyl)thio)-3-phenyl-[1,2,4]triazolo[3,4-b][1,3,4]thiadiazole (**6d**)

Brown solid; Yield: 77%; MP = 199–201 °C; IR (KBr, v_max_, 3035 (CH Aromatic), 2965 (CH Aliphatic), 1451,1436 (C=N) Cm^−1^; ^1^H NMR (250 MHz, DMSO-d_6_) δ 8.15 (d, *J* = 7.30 Hz, 2H, H_2_, H_6_), 7.65–7.46 (m, 5H, H Ar), 7.17 (t, *J* = 8.5 Hz, 2H, H_3_,, H_5_), 4.64 (s, 2H, CH_2_), ppm. ^13^C NMR (62 MHz, DMSO-d_6_): δ 133.72, 132.78, 132.65, 131.79, 130.59, 127.27, 126.79, 117.08, 116.74, 38.20 ppm; ESI–MS (C_16_H_11_FN_4_S_2_): calculated m/z 342.04 [M+H]^+^, observed m/z 342.09 [M+H]^+^; Anal. Calcd C_16_H_11_FN_4_S_2_, C, 56.12; H, 3.24; N, 16.36; Found; C, 56.32; H, 3.45; N, 16.57.

#### 6-((2-chlorobenzyl)thio)-3-phenyl-[1,2,4]triazolo[3,4-b][1,3,4]thiadiazole (**6e**)

Brown solid; Yield: 72%; MP = 224–226 °C; IR (KBr, v_max_, 3030 (CH Aromatic), 2940 (CH Aliphatic), 1456,1434 (C=N) Cm^−1^; ^1^H NMR (250 MHz, DMSO-d_6_) δ 8.15 (d, *J* = 8.20 Hz, 2H, H_2_, H_6_), 7.72–7.53 (m, 4H, H Ar), 7.50–7.44 (m, 1H, H Ar), 7.42–7.29 (m, 2H, H Ar), 4.65 (s, 2H, CH_2_), ppm. ^13^C NMR (62 MHz, DMSO-d_6_): δ 168.36, 157.68, 146.49, 143.04, 139.25, 137.48, 135.29, 134.41, 132.06, 131.25, 130.90, 129.26, 128.93, 128.78, 128.59, 128.43, 127.56, 127.43, 127.19, 126.62, 125.23, 122.92121.36, 115.09, 36.73 ppm; ESI–MS (C_16_H_11_ClN_4_S_2_): calculated m/z 358.01 [M+H]^+^, observed m/z 358.013 [M+H]^+^; Anal. Calcd C_16_H_11_ClN_4_S_2_, C, 53.55; H, 3.09; N, 15.61; Found; C, 53.72; H, 3.14; N, 15.81.

#### 6-((3-chlorobenzyl)thio)-3-phenyl-[1,2,4]triazolo[3,4-b][1,3,4]thiadiazole (**6f**)

Brown solid; Yield: 85%; MP = 216–218 °C; IR (KBr, v_max_, 3025 (CH Aromatic), 2950 (CH Aliphatic), 1456,1434 (C=N) Cm^−1^; ^1^H NMR (250 MHz, DMSO-d_6_) δ 8.16 (d, *J* = 6.60 Hz, 2H, H_2_, H_6_), 7.67–7.48 (m, 5H, H Ar), 7.37–7.29 (m, 2H, H Ar), 4.74 (s, 2H, CH_2_), ppm. ^13^C NMR (62 MHz, DMSO-d_6_): δ 168.36, 157.68, 146.49, 143.04, 139.25, 137.48, 135.29, 134.41, 132.06, 131.25, 130.90, 129.26, 128.93, 128.78, 128.59, 128.43, 127.56, 127.43, 127.19, 126.62, 125.23, 122.92121.36, 115.09, 36.73 ppm; ESI–MS (C_16_H_11_ClN_4_S_2_): calculated m/z 358.01 [M+H]^+^, observed m/z 358.06 [M+H]^+^; Anal. Calcd C_16_H_11_ClN_4_S_2_, C, 53.55; H, 3.09; N, 15.61; Found; C, 53.70; H, 3.10; N, 15.75.

#### 6-((4-chlorobenzyl)thio)-3-phenyl-[1,2,4]triazolo[3,4-b][1,3,4]thiadiazole (**6g**)

Brown solid; Yield: 87%; MP = 218–220 °C; IR (KBr, v_max_, 3020 (CH Aromatic), 2970 (CH Aliphatic), 1457,1432 (C=N) Cm^−1^; ^1^H NMR (250 MHz, DMSO-d_6_) δ 8.13 (d, *J* = 7.30 Hz, 2H, H_2_, H_6_), 7.64–7.47 (m, 5H, H Ar), 7.41 (d, *J* = 5.90 Hz, 2H, H_3_,, H_5_), 4.63 (s, 2H, CH_2_), ppm. ^13^C NMR (62 MHz, DMSO-d_6_): δ 136.67, 132.45, 131.80, 130.58, 130.02, 127.26, 126.75, 38,21 ppm; ESI–MS (C_16_H_11_ClN_4_S_2_): calculated m/z 358.01 [M+H]^+^, observed m/z 358.03 [M+H]^+^; Anal. Calcd C_16_H_11_ClN_4_S_2_, C, 53.55; H, 3.09; N, 15.61; Found; C, 53.75; H, 3.15; N, 15.80.

#### 6-((3,4-dichlorobenzyl)thio)-3-phenyl-[1,2,4]triazolo[3,4-b][1,3,4]thiadiazole (**6h**)

Brown solid; Yield: 81%; MP = 215–217 °C; IR (KBr, v_max_, 3065 (CH Aromatic), 2950 (CH Aliphatic), 1459,1437 (C=N) Cm^−1^; ^1^H NMR (250 MHz, DMSO-d_6_) δ 8.11 (d, *J* = 7.30 Hz, 2H, H_2_, H_6_), 7.78 (d, *J* = 5.90 Hz, 2H, H_6_), 7.62–7.43 (m, 5H, H Ar), 4.60 (s, 2H, CH_2_), ppm. ^13^C NMR (62 MHz, DMSO-d_6_): δ 139.11, 132.14, 131.85, 131.74, 130.83, 130.55, 127.23, 126.77, 37.60 ppm; ESI–MS (C_16_H_10_Cl_2_N_4_S_2_): calculated m/z 391.97 [M+H]^+^, observed m/z 391.99 [M+H]^+^; Anal. Calcd C_16_H_10_Cl_2_N_4_S_2_, C, 48.86; H, 2.56; N, 14.24; Found; C, 49.05; H, 2.75; N, 14.45;.

#### 6-((2-bromobenzyl)thio)-3-phenyl-[1,2,4]triazolo[3,4-b][1,3,4]thiadiazole (**6i**)

Brown solid; Yield: 89%; MP = 210–212 °C; IR (KBr, v_max_, 3020 (CH Aromatic), 2885 (CH Aliphatic), 1450,1431 (C=N) Cm^−1^; ^1^H NMR (250 MHz, DMSO-d_6_) δ 8.16 (d, *J* = 6.50 Hz, 2H, H_2_, H_6_), 7.70–7.49 (m, 5H, H Ar), 7.35 (d, *J* = 7.30 Hz, 1H, H Ar), 7.27 (d, *J* = 8.00 Hz, 1H, H_2_, Ar), 4.72 (s, 2H, CH_2_), ppm. ^13^C NMR (62 MHz, DMSO-d_6_): δ 133.29, 132.12, 131.76, 130.61, 129.64, 127.25, 126.77, 38.09 ppm; ESI–MS (C_16_H_11_BrN_4_S_2_): calculated m/z 401.96 [M+H]^+^, observed m/z 401.99 [M+H]^+^; Anal. Calcd C_16_H_11_Br_4_S_2_, C, 47.65; H, 2.75; N, 13.89; Found; C, 47.80; H, 2.92; N, 14.10.

#### 6-((3-bromobenzyl)thio)-3-phenyl-[1,2,4]triazolo[3,4-b][1,3,4]thiadiazole (**6j**)

Brown solid; Yield: 75%; MP = 225–227 °C; IR (KBr, v_max_, 3060 (CH Aromatic), 2950 (CH Aliphatic), 1457,1433 (C=N) Cm^−1^; ^1^H NMR (250 MHz, DMSO-d_6_) δ 8.14 (d, *J* = 7.30 Hz, 2H, H_2_, H_6_), 7.75 (s, 1H, H_2_), 7.63–7.42 (m, 5H, H _Ar_), 7.11 (t, *J* = 7.50 Hz, 1H, H_5_), 4.64 (s, 2H, CH_2_), ppm. ^13^C NMR (62 MHz, DMSO-d_6_): δ 131.80, 130.56, 127.24, 125.10, 71.23, 38.11. ppm; ESI–MS (C_16_H_11_BrN_4_S_2_): calculated m/z 342.04 [M+H]^+^, observed m/z 342.15 [M+H]^+^; Anal. Calcd C_16_H_11_BrN_4_S_2_ C, 47.65; H, 2.75; N, 13.89; Found; C, 47.79; H, 2.89; N, 14.13.

#### 6-((4-bromobenzyl)thio)-3-phenyl-[1,2,4]triazolo[3,4-b][1,3,4]thiadiazole (**6k**)

Brown solid; Yield: 78%; MP = 212–214 °C; IR (KBr, v_max_, 3060 (CH Aromatic), 2970 (CH Aliphatic), 1456,1433 (C=N) Cm^−1^; ^1^H NMR (250 MHz, DMSO-d_6_) δ 8.12 (d, *J* = 6.50 Hz, 2H, H_2_, H_6_), 7.64–7.50 (m, 5H, H Ar), 7.45 (d, *J* = 8.20 Hz, 2H, H_3_, H_5_), 4.61 (s, 2H, CH_2_), ppm. ^13^C NMR (62 MHz, DMSO-_d6)_: δ 137.09, 132.94, 132.75, 131.79, 130.57, 127.25, 126.74, 122.43, 38.25 ppm; ESI–MS (C_16_H_11_BrN_4_S_2_): calculated m/z 401.96 [M+H]^+^, observed m/z 401.98 [M+H]^+^; Anal. Calcd C_16_H_11_BrN_4_S_2_, C, 47.65; H, 2.75; N, 13.89; Found; C, 47.80; H, 2.92; N, 14.10.

#### 6-((4-nitrobenzyl)thio)-3-phenyl-[1,2,4]triazolo[3,4-b][1,3,4]thiadiazole (**6k**)

Yellow solid; Yield: 79%; MP = 220–222 °C; IR (KBr, v_max_, 3048 (CH Aromatic), 2863 (CH Aliphatic), 1454,1432 (C=N) Cm^−1^; ^1^H NMR (250 MHz, DMSO-d_6_) δ 8.20 (d, J = 6.3 Hz, 2H, H_2_, H_6_), 8.10 (d, J = 8.3 Hz, 2H, H_2_, H_6_), 7.79 (d, J = 8.7 Hz, 2H, H_3_, H_5_), 7.57 (m, 3H, H_Ar_). 4.78 (s, 2H, CH_2_) ppm. ^13^C NMR (62 MHz, DMSO-d6): δ 131.80, 130.56, 127.24, 125.10, 71.23, 38.01 ppm; ESI–MS (C_16_H_11_N_5_O_2_S_2_): calculated m/z 401.96 [M+H]^+^, observed m/z 401.99 [M+H]^+^; Anal. Calcd C_16_H_11_N_5_O_2_S_2_, C, 52.02; H, 3.00; N, 18.96; Found; C, 52.27; H, 3.19; N, 19.14.

#### 6-((2-methylbenzyl)thio)-3-phenyl-[1,2,4]triazolo[3,4-b][1,3,4]thiadiazole (**6l**)

Brown solid; Yield: 83%; MP = 212–214 °C; IR (KBr, v_max_, 3035 (CH Aromatic), 2965 (CH Aliphatic), 1452,1433 (C=N) Cm^−1^; ^1^H NMR (250 MHz, DMSO-d_6_) δ 8.16 (d, *J* = 7.10 Hz, 2H, H_2_, H_6_), 7.64–7.45 (m, 3H, H Ar), 7.26 (d, *J* = 7.60 Hz, 1H, H_3_), 7.12–7.02 (m, 2H, H Ar), 6.84 (d, *J* = 7.90 Hz, 1H, H_2_), 4.60 (s, 2H, CH_2_), 3.62 (s, 3H, CH_3_) ppm. ^13^C NMR (62 MHz, DMSO-d_6_): δ 131.77, 131.17, 130.57, 127.27, 126.78, 122.79, 116.25, 114.85, 56.49, 38.89 ppm; ESI–MS (C_17_H_14_N_4_S_2_): calculated m/z 338.07 [M+H]^+^, observed m/z 338.13 [M+H]^+^; Anal. Calcd C_17_H_14_N_4_S_2_, C, 60.33; H, 4.17; N, 16.55; Found; C, 60.55; H, 4.38; N, 16.74.

#### 6-((3-methylbenzyl)thio)-3-phenyl-[1,2,4]triazolo[3,4-b][1,3,4]thiadiazole (**6m**)

Brown solid; Yield: 86%; MP = 213–215 °C; IR (KBr, v_max_, 3020 (CH Aromatic), 2975 (CH Aliphatic), 1454,1436 (C=N) Cm^−1^; ^1^H NMR (250 MHz, DMSO-d_6_) δ 8.17 (d, *J* = 6.60 Hz, 2H, H_2_, H_6_), 7.63–7.52 (m, 3H, H Ar), 7.45 (d, *J* = 6.60 Hz, 1H, H Ar), 7.26–7.10 (m, 3H, H Ar), 4.67 (s, 2H, CH_2_), 2.36 (s, 3H, CH_3_) ppm. ^13^C NMR (62 MHz, DMSO-d_6_): δ 132.01, 131.79, 131.70, 130.58, 129.82, 127.62, 127.62, 127.31, 126.83, 37.62, 20.31 ppm; ESI–MS (C_17_H_14_N_4_S_2_): calculated m/z 338.07 [M+H]^+^, observed m/z 338.15 [M+H]^+^; Anal. Calcd C_17_H_14_N_4_S_2_, C, 60.33; H, 4.17; N, 16.55; Found; C, 60.53; H, 4.35; N, 16.75.

#### 6-(methylthio)-3-phenyl-[1,2,4]triazolo[3,4-b][1,3,4]thiadiazole (**6n**)

Brown solid; Yield: 81%; MP = 213–215 °C; IR (KBr, v_max_, 3010 (CH Aromatic), 2960 (CH Aliphatic), 1456,1431 (C=N) Cm^−1^; ^1^H NMR (250 MHz, DMSO-d_6_) δ 8.17 (d, *J* = 7.50 Hz, 2H, H_2_, H_6_), 7.62–7.42 (m, 3H, H Ar), 2.81 (s, 2H, CH_3_), ppm. ^13^C NMR (62 MHz, DMSO-d_6_): δ ^13^C NMR (63 MHz, DMSO) δ 131.72, 130.58, 127.24, 17.26.ppm; ESI–MS (C_10_H_8_N_4_S_2_): calculated m/z 248. 02 [M+H]^+^, observed m/z 248.11 [M+H]^+^; Anal. Calcd C_10_H_8_N_4_S_2_, C, 48.37; H, 3.25; N, 22.56; Found; C, 48.57; H, 3.42; N, 22.49.

### Screening of urease inhibitory activities

Urease inhibition effects of the synthesized compounds were determined according to the previously reported procedure^37–39^. Briefly, 100 μL of the synthesized compounds at different concentrations was added to 850 μL of urea as substrate and 50 μL urease (3 mg/mL) in phosphate buffer, pH = 7.4). After 30 min, to 100 μL of the incubated solution, 500 μL of solution I (5.0 g phenol and 25.0 mg sodium nitroprusside in 500 mL water) was added, followed by the addition of 500 μL of solution II (2.5 g sodium hydroxide, 4.2 mL sodium hypochlorite, and 5% chlorine in 500 mL water) which was further incubated at 37 °C for 30 min. The absorbance of blue-colored indophenol of each cell is related to the percentage of enzyme inhibition using the following equation at 625 nm. The IC_50_ values for all synthesized compounds were calculated using GraphPad Prism software (GraphPad Software, Inc., San Diego, CA).

### Kinetic studies

The kinetic study for the inhibition of urease by compound **6a** was carried out using four different concentrations of inhibitor. Compound 6a was tested at 0, 0.5, 1, and 28 μM concentrations against urease for the kinetic study. The Lineweaver–Burk reciprocal plot was constructed by plotting 1/V against 1/[S] at variable concentrations of the substrate urea (3.12 to 100 mM). The inhibition constant *K*_*i*_ was calculated by the plot of slopes versus the corresponding concentrations of compound **6a**.

### In silico studies

Maestro Molecular Modeling platform (version 10.5) by Schrödinger, LLC (*Maestro, Schrödinger, LLC, New York, NY, 2021*) was used to perform the docking study of compound **6a** and thiourea on the jack bean urease enzyme. The corresponding crystallographic structure of jack bean urease was downloaded from http://www.rcsb.com by the PDB (PDB ID:4h9m). preparation wizard^40^. Afterward, missing used for primary preparation of the receptor for the next stage, missed sidechains and loops were filled utilizing the prime tool^41^. 2D structure of ligands sketched in ChemDraw (ver. 16) and saved as SDF files. The Ligprep module (*LigPrep, Schrödinger, LLC, New York, NY, 2021*) was used to prepare ligand molecules with OPLS_2005 forcefield and EPIK program^42^ at a target pH of 7.0 ± 1. Induced fit docking simulation^43^ was performed considering AHA as the grid center, with a maximum number of 20 poses for each ligand. Receptor and ligand van der Waals radii were set as 0.7 A and 0.5 Å, respectively. Structures with prime energy levels beyond 30 kcal/mol were eliminated based on standard precision glide docking. Molecular dynamic simulation was performed using maestro desmond^44^. MD simulation complexes obtained from the stage IFD results. The simulation was conducted in a cubic cell filled with 27869 TI3P model water molecules. 92 sodium atoms and 78 chloride ions were added to neutralize the system electrostatic charge. The NPT ensemble (constant number of atoms; constant pressure, i.e., 1.01325 bar; and constant temperature, i.e.,300 K) was used with default settings. Overall simulation duration set on 100 ns for both urease-thiourea and urease-compound **6a** complexes with 100 ps for each trajectory frame. The simulation results were analyzed using the maestro graphical interface.

### In vitro antimicrobial activities

The antimicrobial activities of all derivatives against *C. neoformans* (H99), and clinical isolate of *P. mirabilis*as urease positive microorganism, were tested as recommended by the Clinical and Laboratory Standards Institute (CLSI) (M07-A9 for bacteria; M27-A3 for yeasts). The compounds were diluted, and stock solutions of 20 mg/mL in DMSO were prepared. Mueller–Hinton Broth (HiMedia) and RPMI-1640 (Sigma) were prepared as recommended for antimicrobial susceptibility testing of bacterial and fungal strains, respectively. Two-fold dilutions were made in the range of 1–512 μg/mL for tested compounds. The microbroth dilution test was accomplished using a 96-well microtiter plate containing growth control (drug free wellls) and sterility control (only broth media). The antimicrobial susceptibility test was accomplished by adding a cell suspension adjusted to the 0.5 McFarland standard (1–2 × 10^8^ CFU/mL for bacterial strains; 1–5 × 10^6^ cells/mL for yeast) to different concentrations of tested compounds. Following incubation, the minimum inhibitory concentration (MIC) was established as the lowest concentration of compounds that completely inhibits the organism's growth in wells, as detected visually. All experiments were performed in duplicates.

### Anti-ureolytic activities against ureolytic microorganisms

The colorimetric microdilution technique using urea broth media (Merck) was used to examine the ureolytic activity of *C. neoformans* (H99), and clinical isolate of *P. mirabilis* treated with tested substances (supplemented with glucose; pH = 6 for *C. neoformans*). Compounds in the concentration range of 1 to 512 μg/mL were exposed to ureolytic microorganisms, and the color of the medium was evaluated visually and spectroscopically at 560 nm after three days for *C. neoformans* and 24 h for *P. mirabilis*. The positive control, which included ureolytic bacteria but no drugs, changed color from yellow to dark pink or magenta. This shifts, allowing the determination of the inhibitory activity of compounds against the urease activity of organisms even without a microliter plate reader^45,46^.

## Supplementary Information

Supplementary Figures.

## Data Availability

The datasets generated and/or analyzed during the current study are available in the Worldwide Protein Data Bank repository with PDB DOI of 10.2210/pdb4H9M/pdb, (https://www.rcsb.org/structure/4h9m).
