# Supplementary material for: [1,2,4]triazolo[3,4-b][1,3,4]thiadiazole derivatives as new therapeutic candidates against urease positive microorganisms: design, synthesis, pharmacological evaluations, and in silico studies
Source: Sci Rep. 2023 Jun 22;13:10136. doi: 10.1038/s41598-023-37203-z (PMC10287669; doi:10.1038/s41598-023-37203-z)

Fig. S1. NMR of **6a**

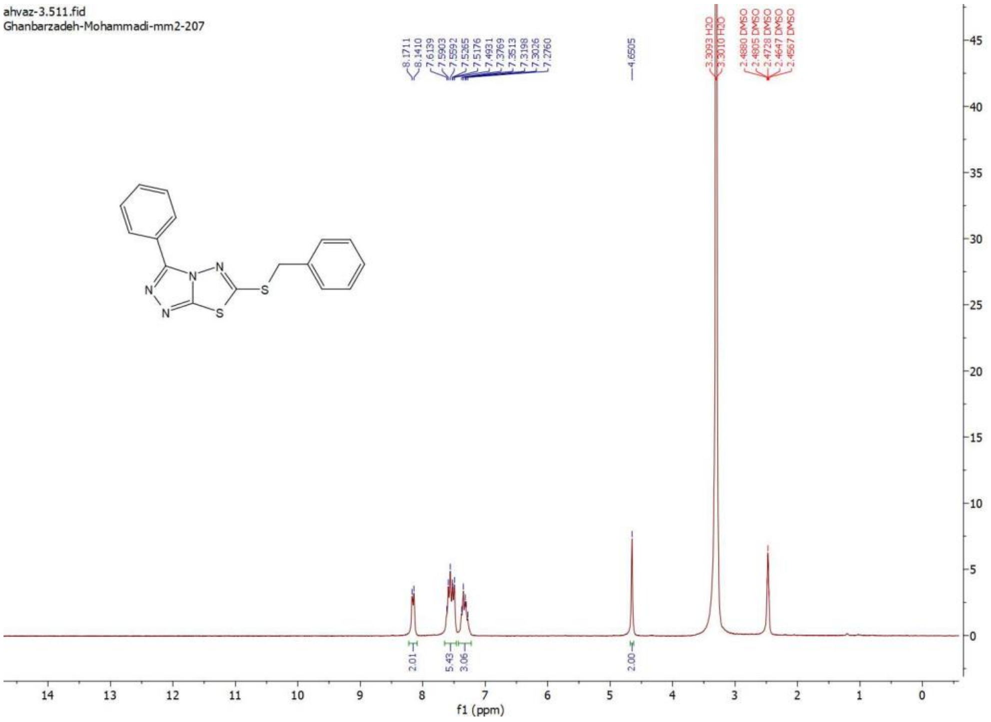


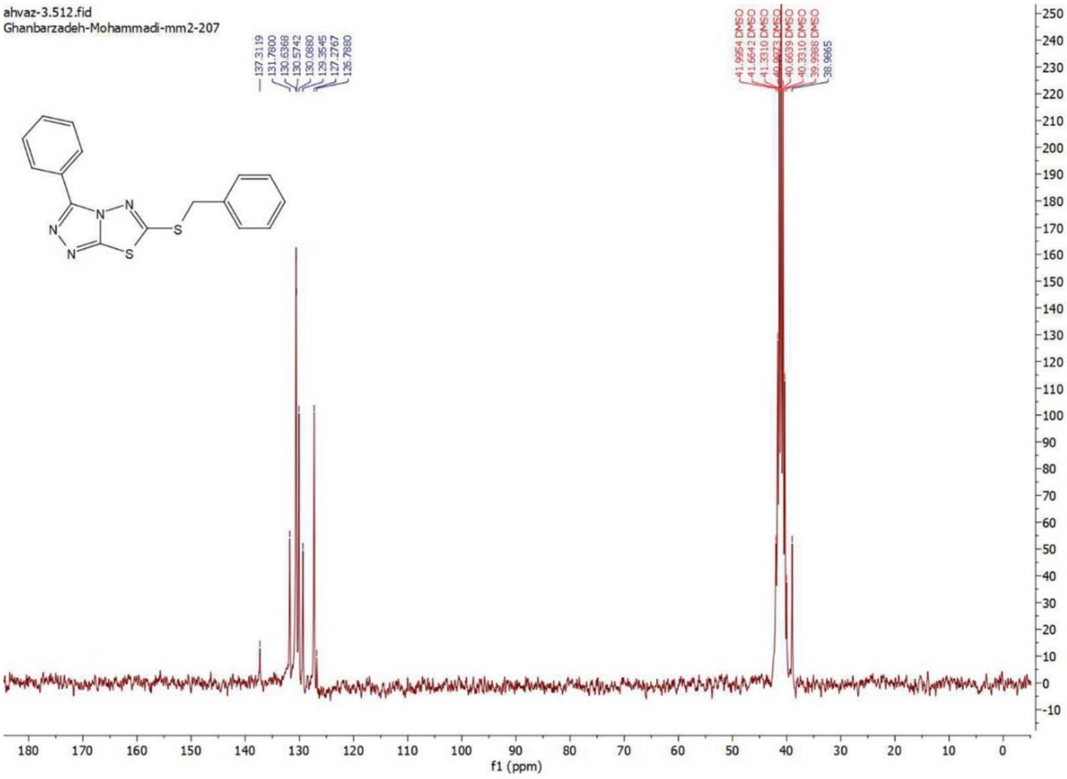

Fig. S2. NMR of **6b**

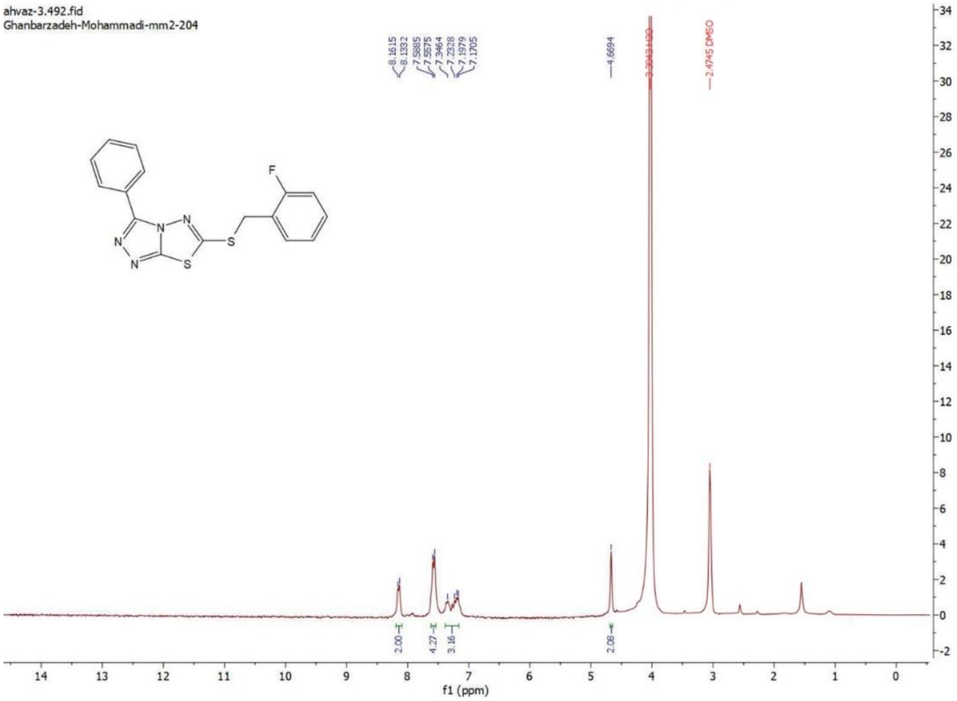


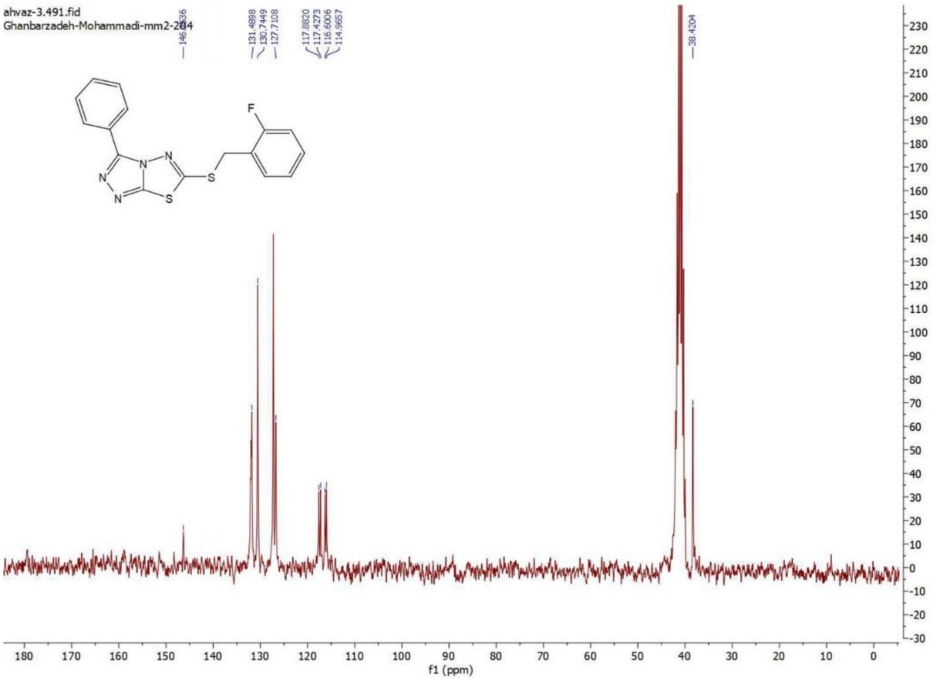

Fig. S3. NMR of **6c**

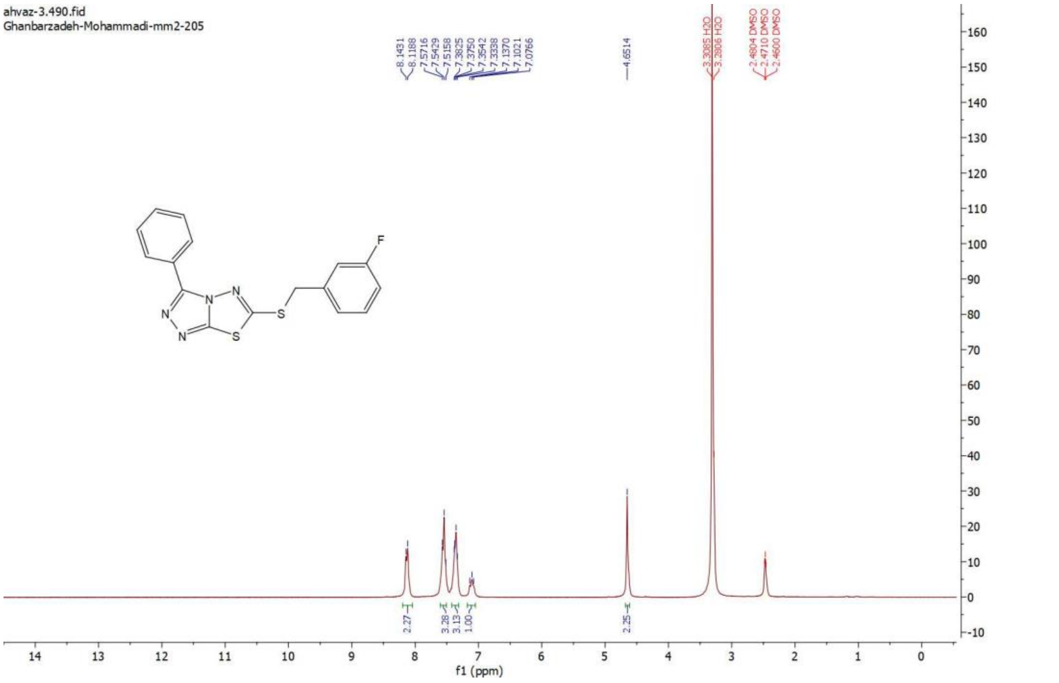


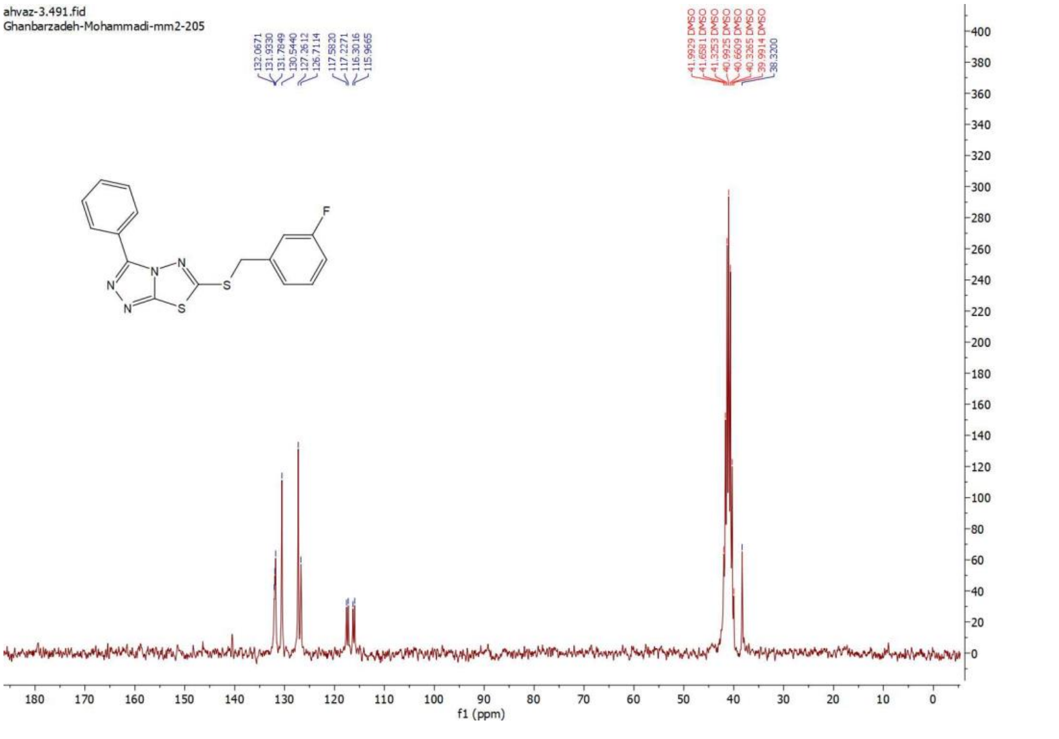

Fig. S4. NMR of **6d**

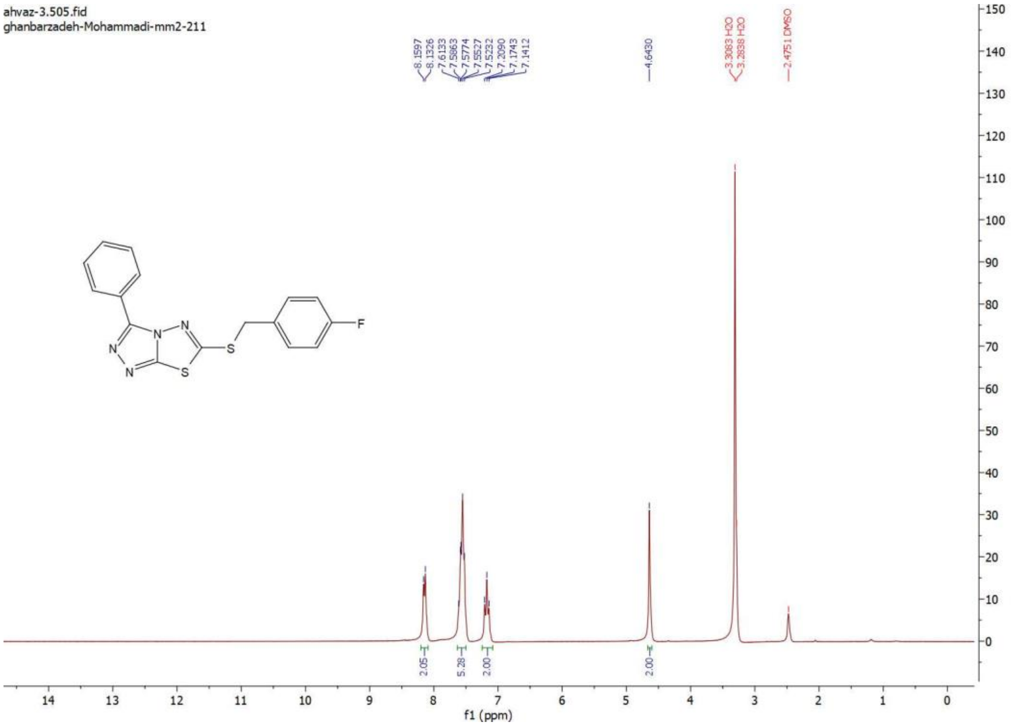


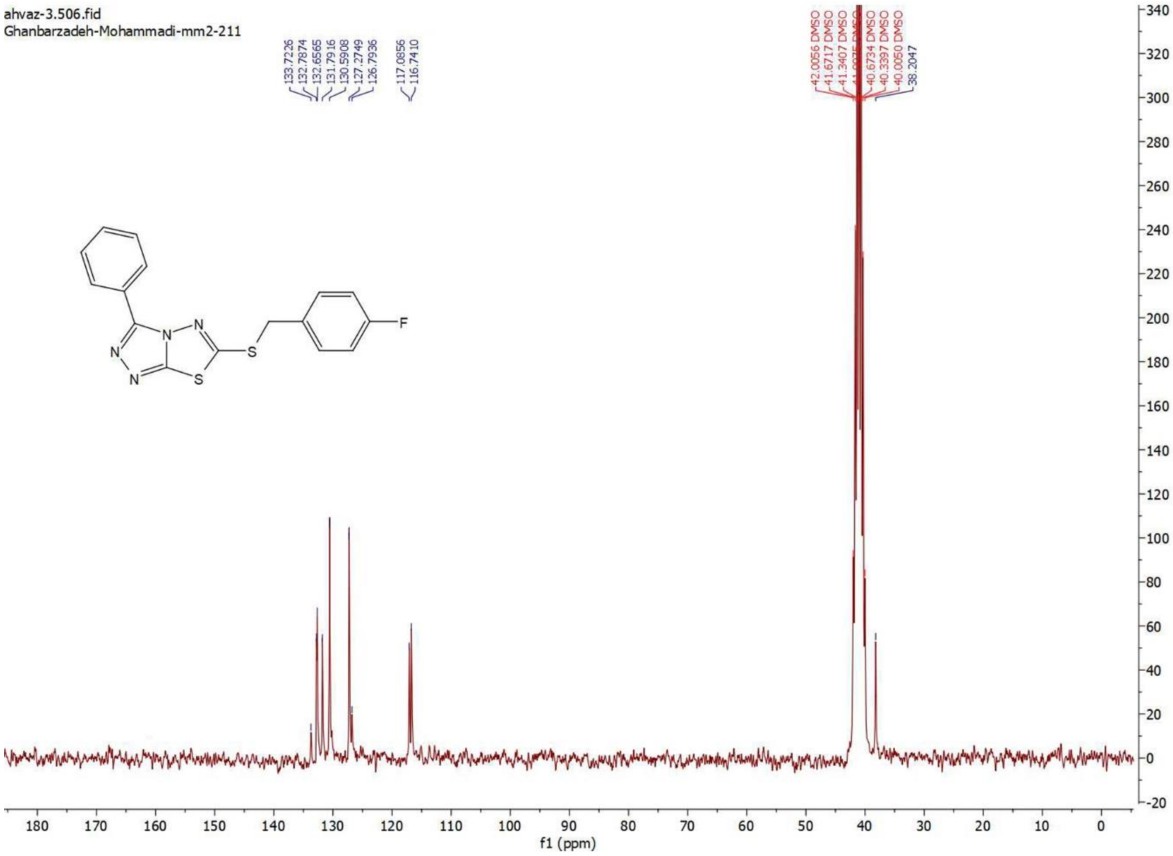

Fig. S5. NMR of **6e**

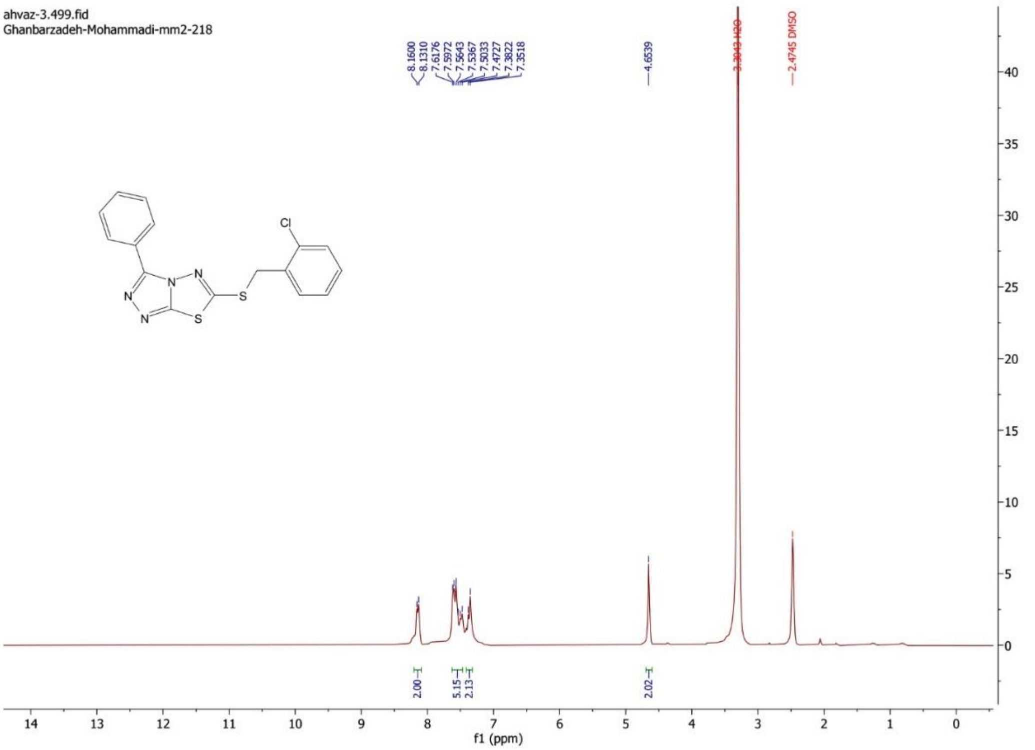


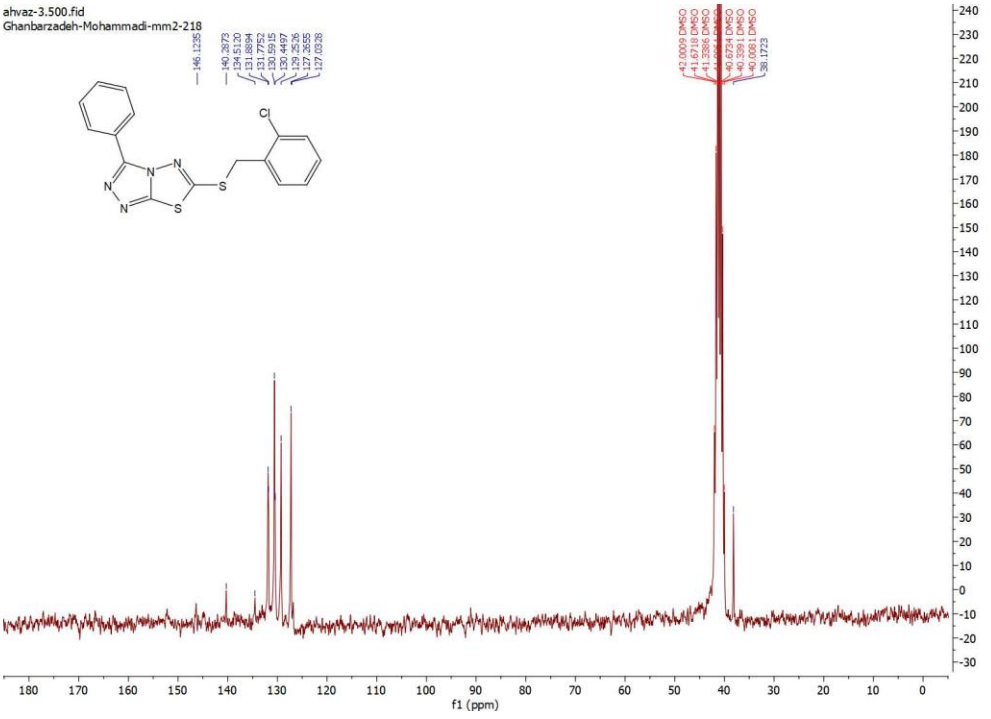

Fig. S6. NMR of **6f**

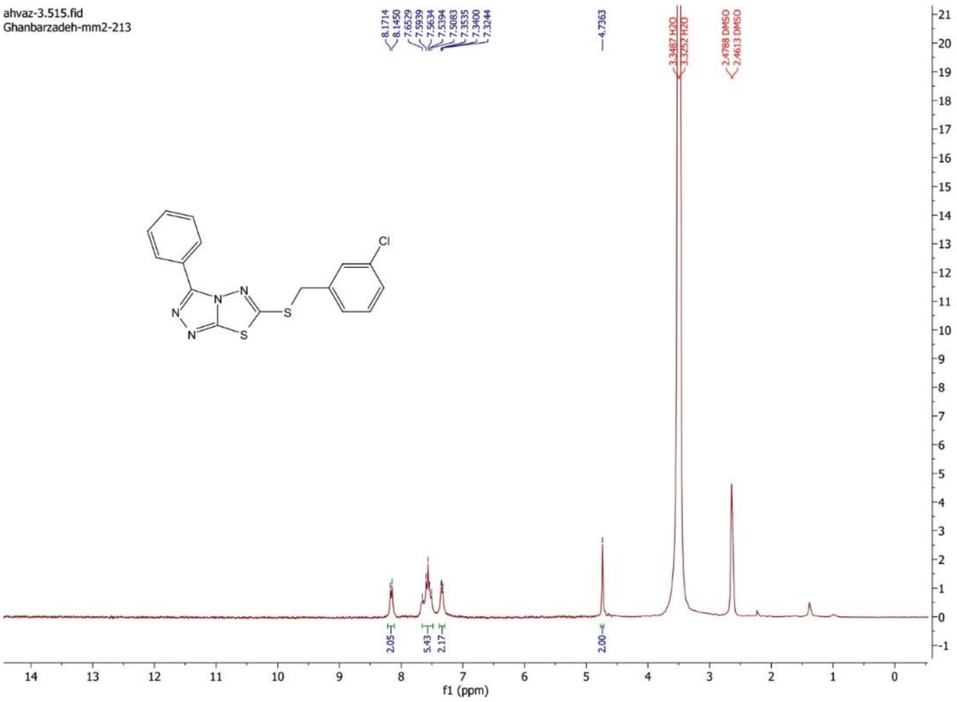


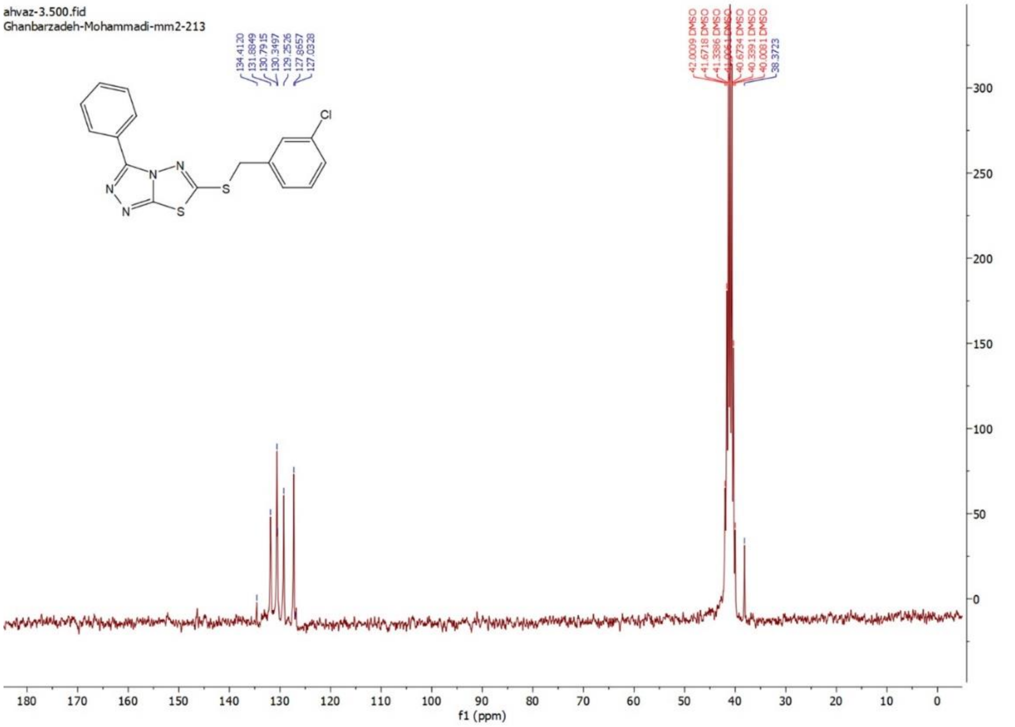

Fig. S7. NMR of **6g**

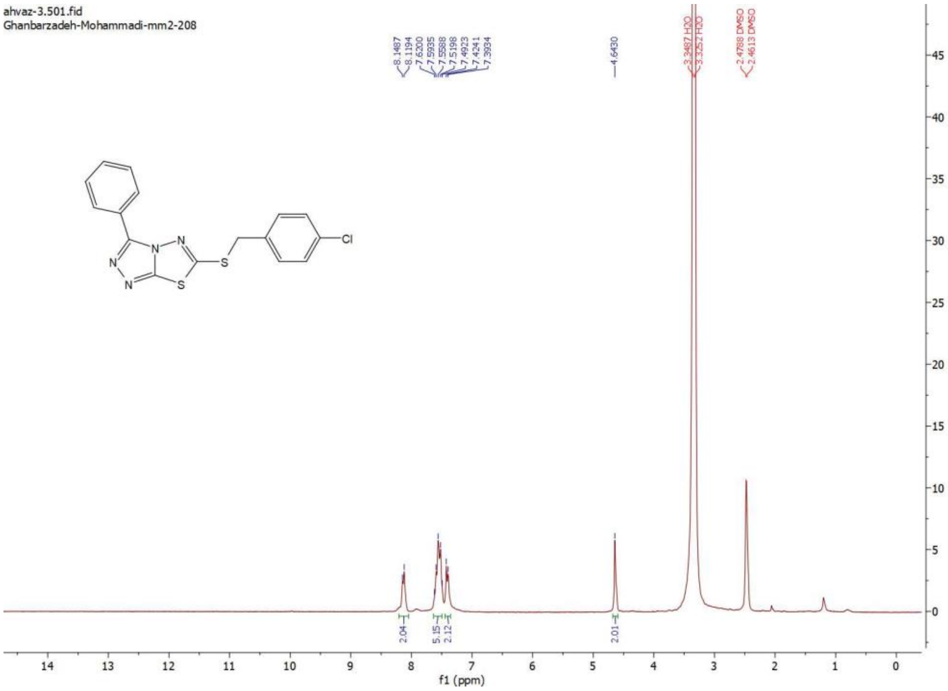


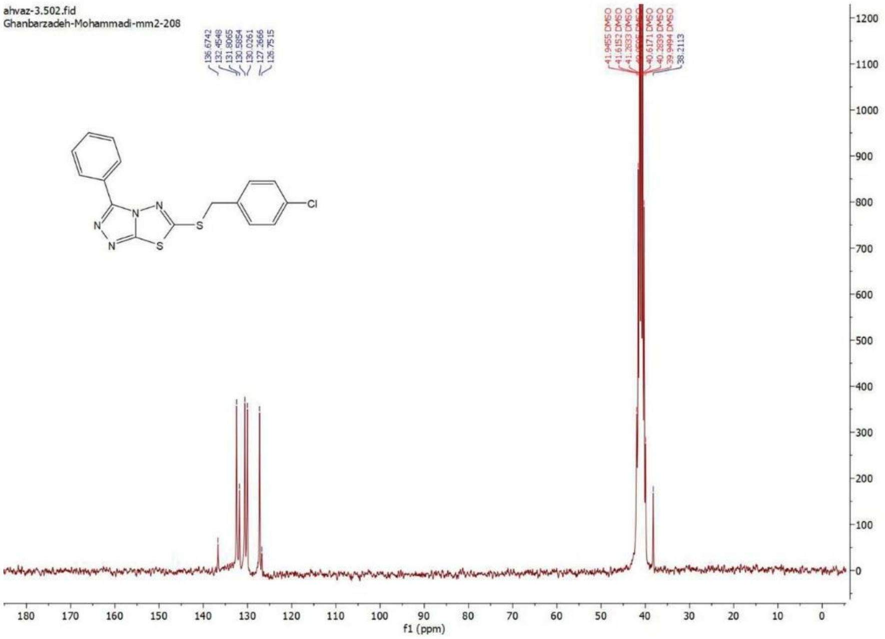

Fig. S8. NMR of **6h**

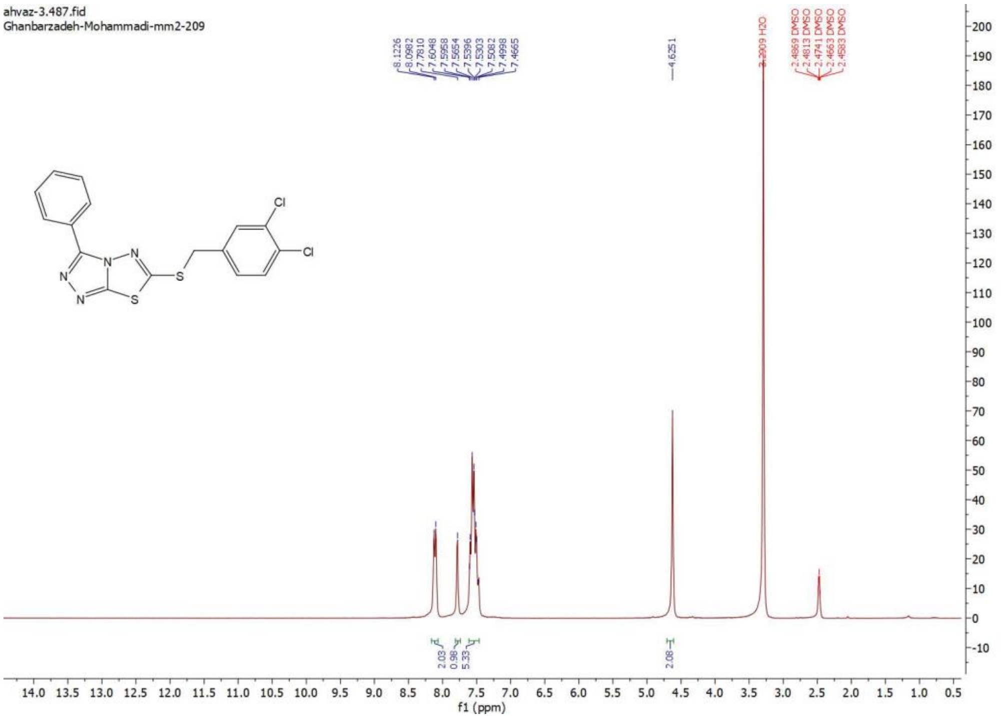


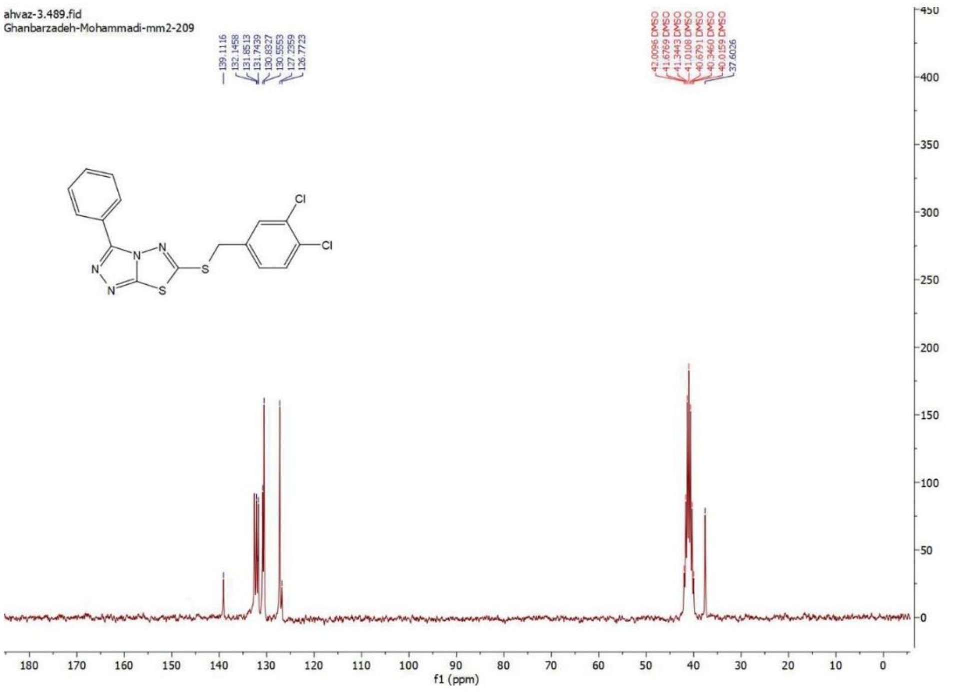

Fig. S9. NMR of **6i**

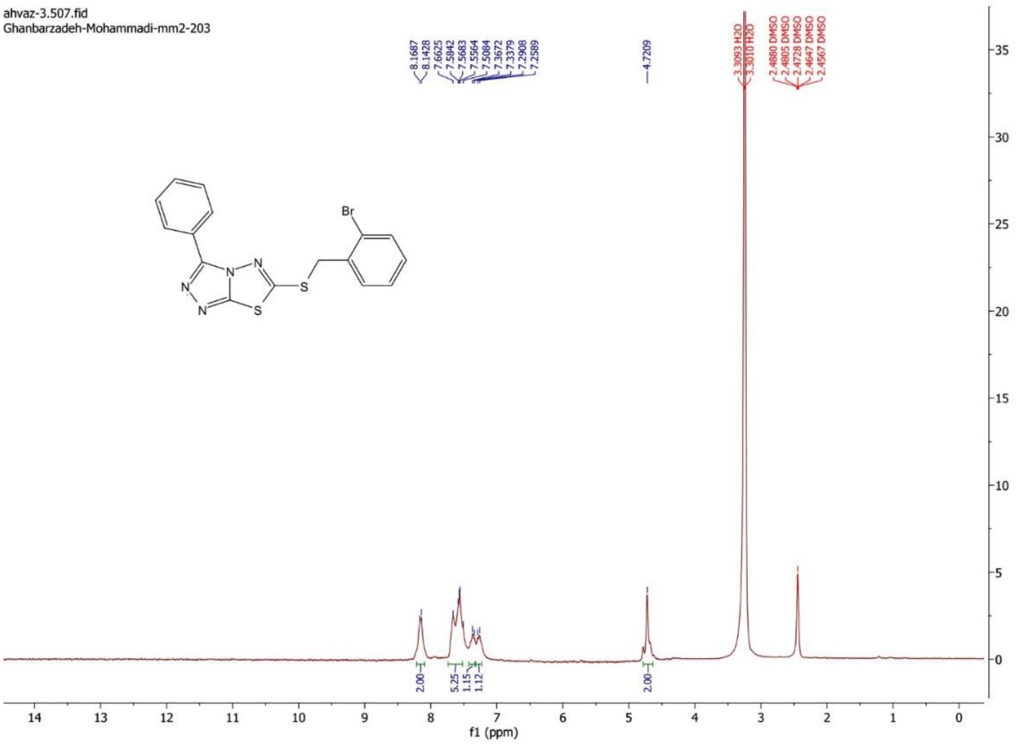


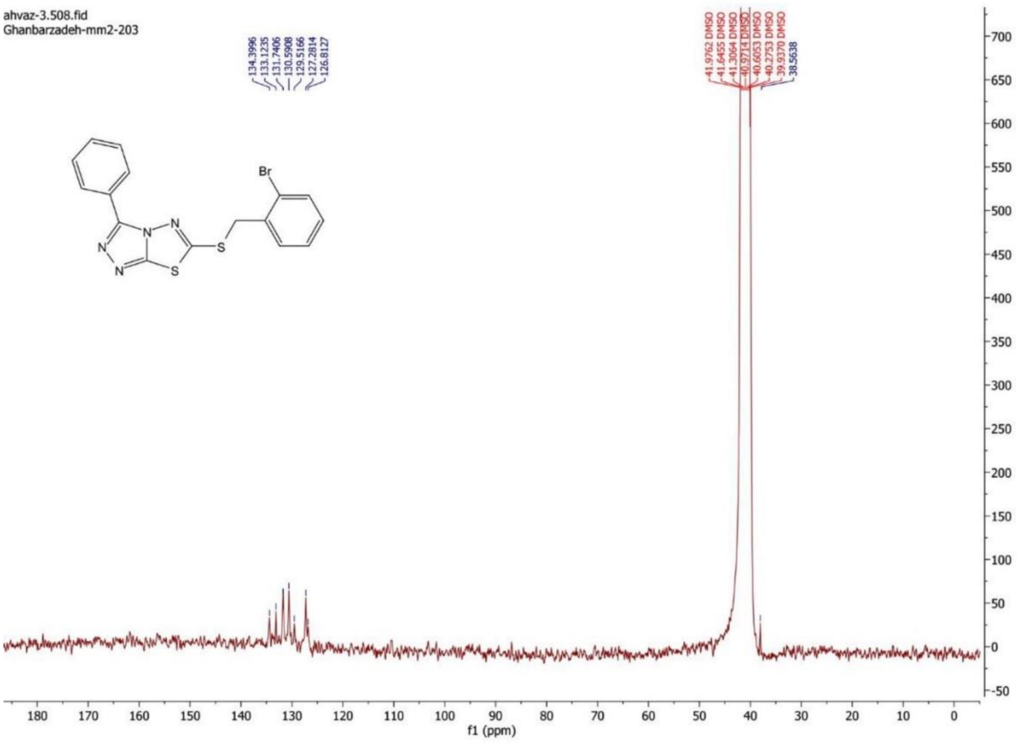

Fig. S10. NMR of **6j**

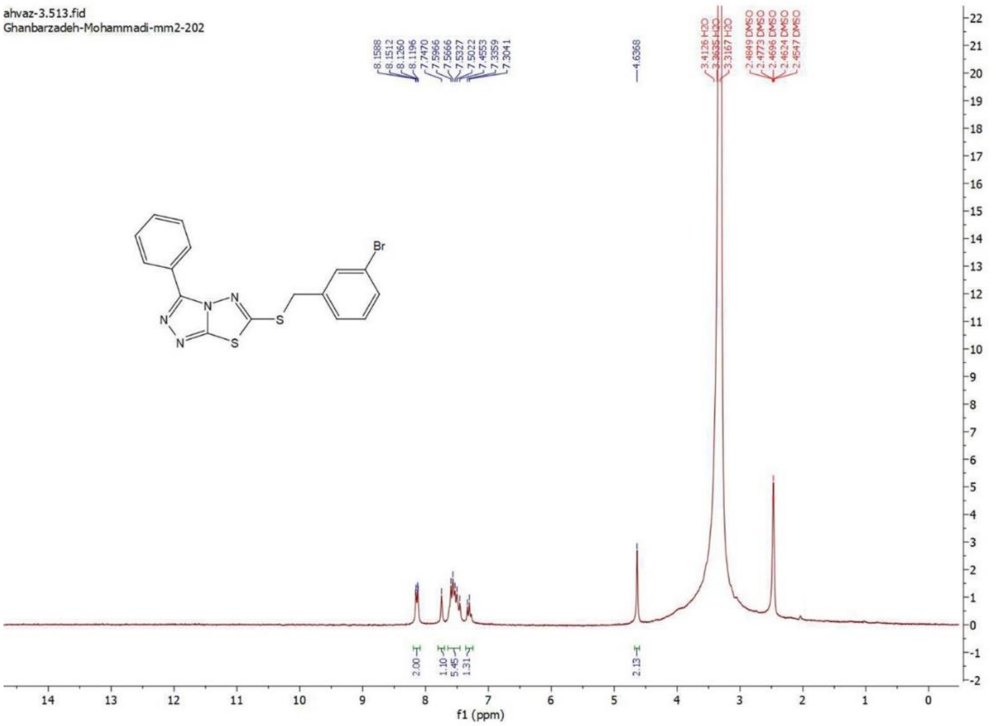

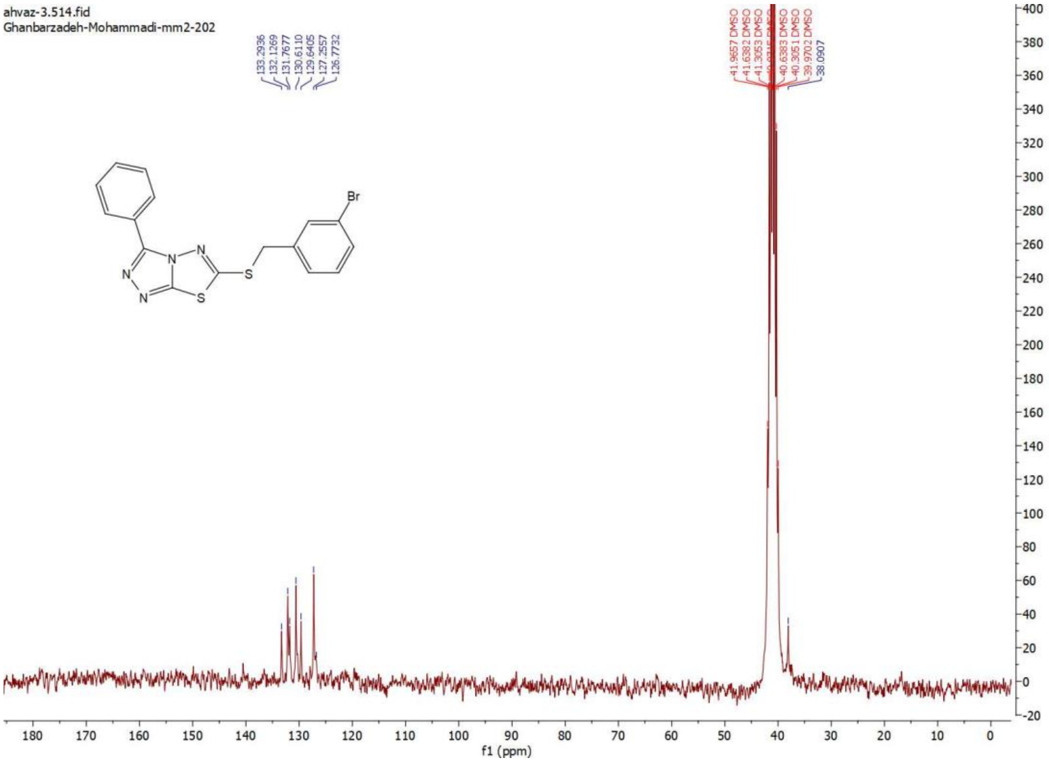

Fig. S11. NMR of **6k**

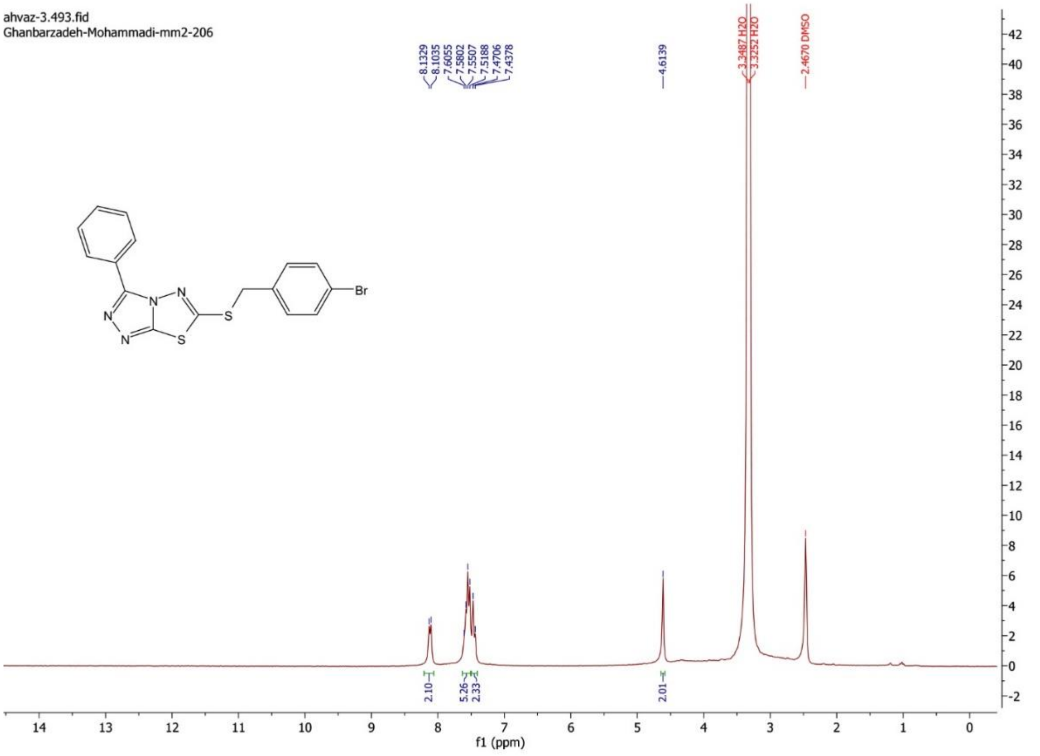


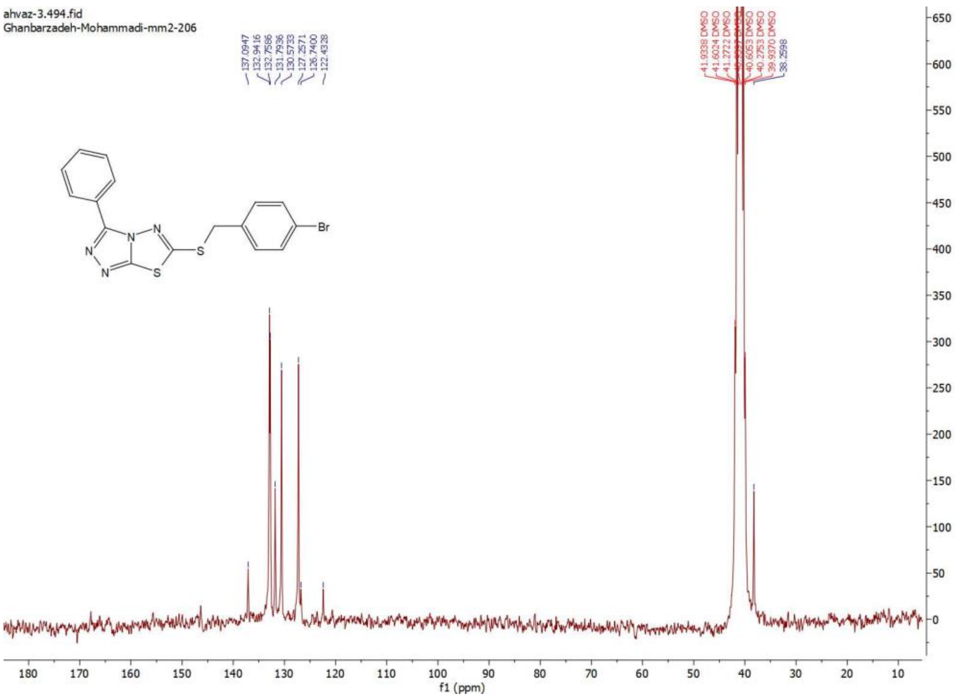

Fig. S12. NMR of **6l**

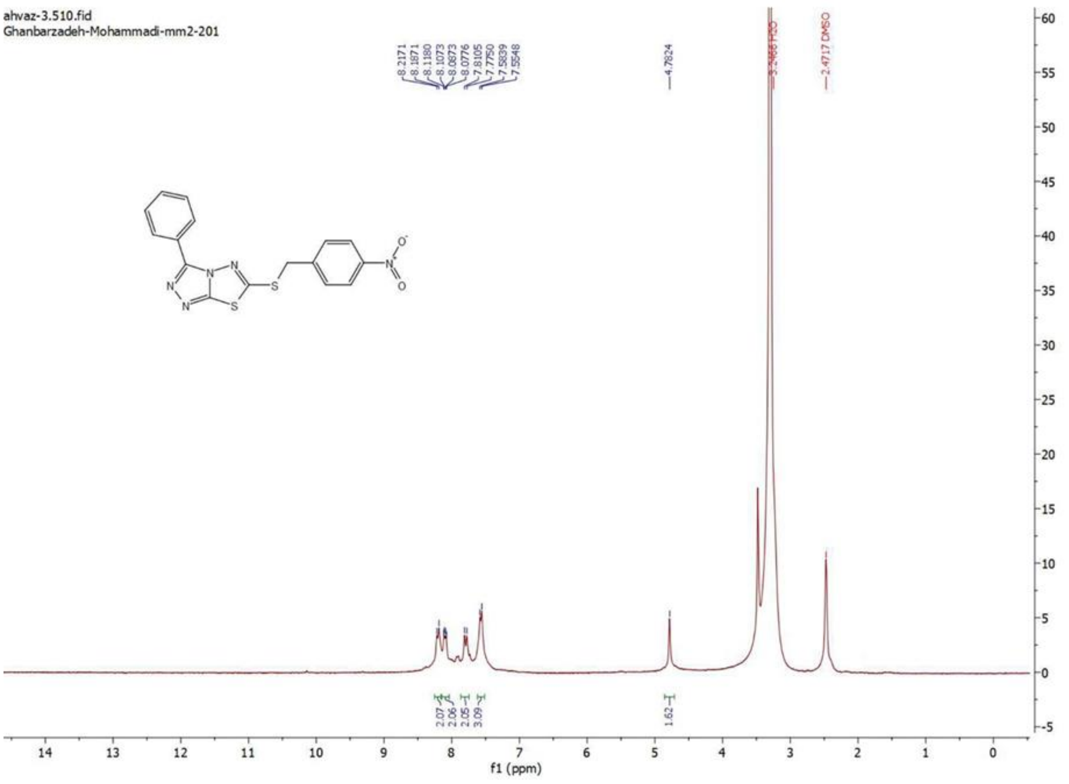

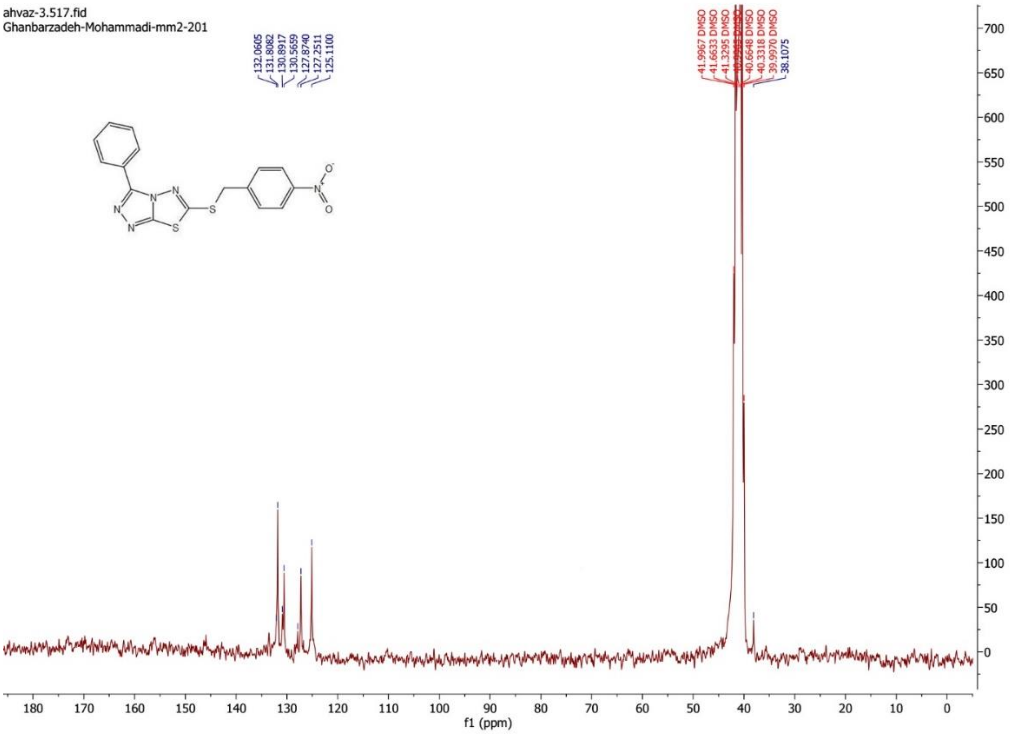

Fig. S13. NMR of **6m**

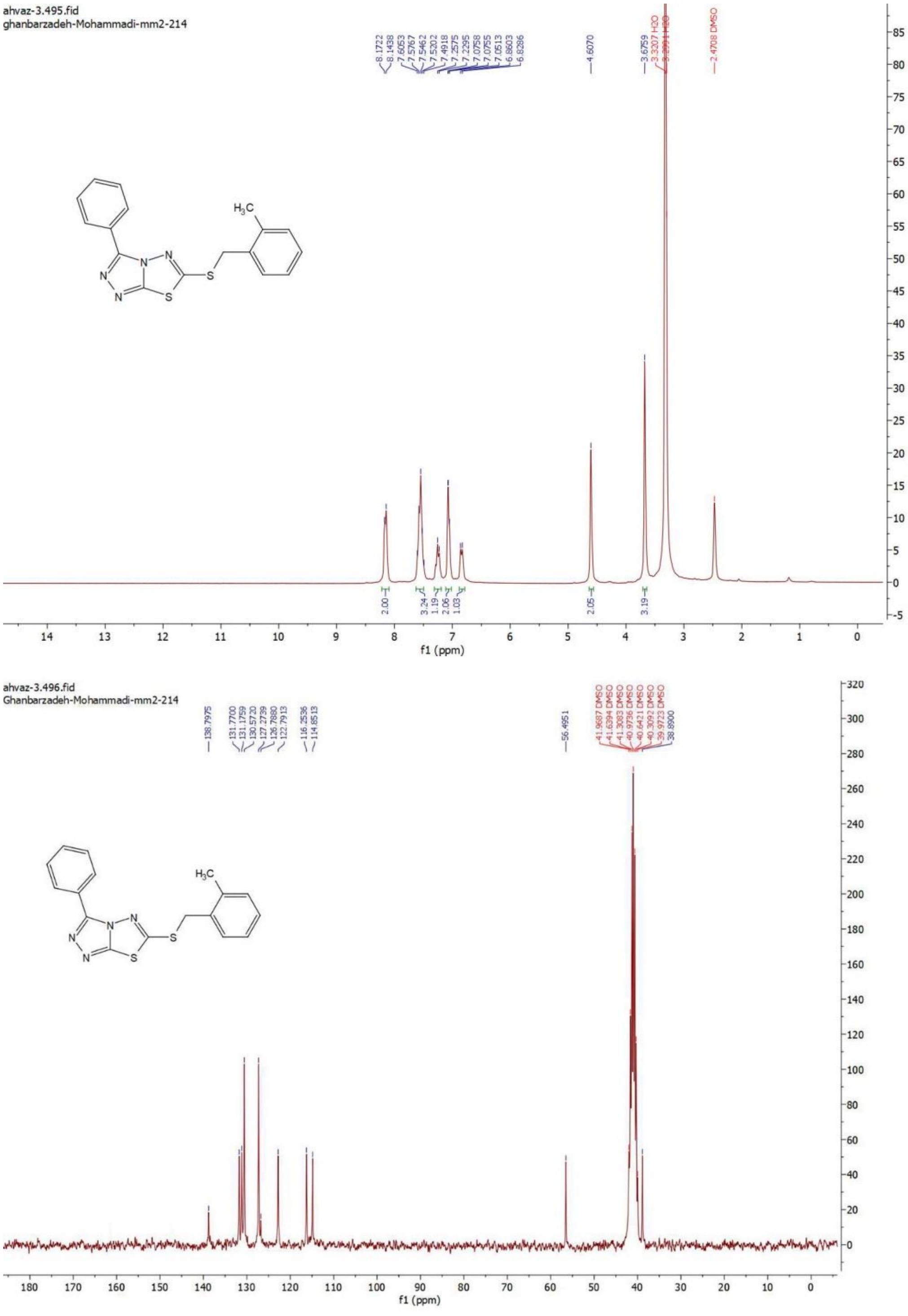

Fig. S14. NMR of **6n**

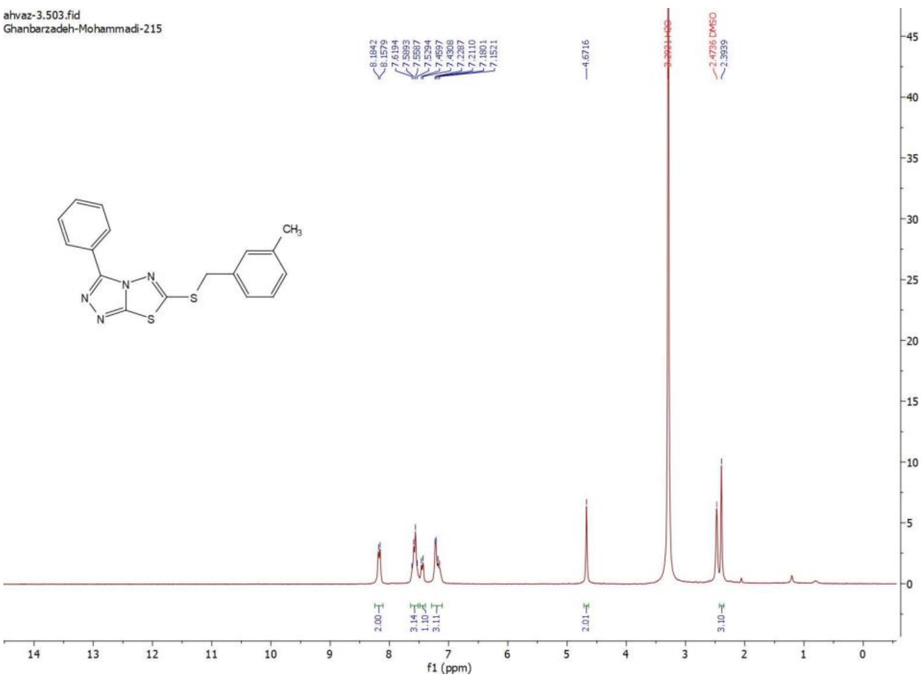


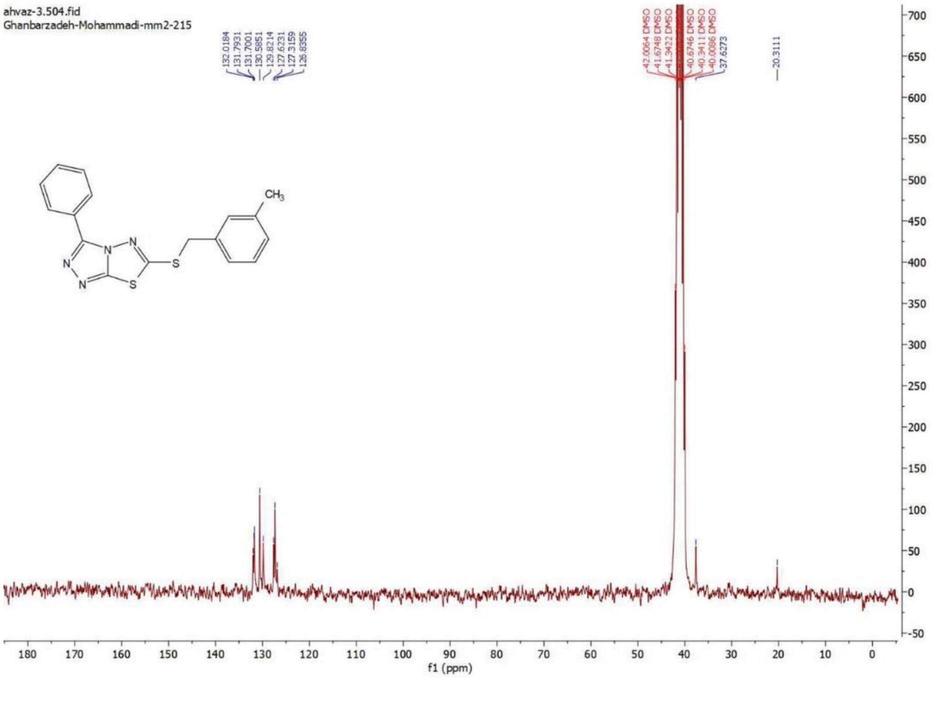

Supplement: Supplementary file 1 — Supplementary Figures. [file 41598_2023_37203_MOESM1_ESM.pdf]
